# Modular Synthesis of Semiconducting Graft Copolymers to Achieve “Clickable” Fluorescent Nanoparticles with Long Circulation and Specific Cancer Targeting

**DOI:** 10.1002/adma.202300413

**Published:** 2023-04-02

**Authors:** Adam Creamer, Alessandra Lo Fiego, Alice Agliano, Lino Prados-Martin, Håkon Høgset, Adrian Najer, Daniel A. Richards, Jonathan P. Wojciechowski, James E. J. Foote, Nayoung Kim, Amy Monahan, Jiaqing Tang, André Shamsabadi, Léa N. C. Rochet, Ioanna A. Thanasi, Laura R. de laBallina, Charlotte L. Rapley, Stephen Turnock, Elizabeth A. Love, Laurence Bugeon, Margaret J. Dallman, Martin Heeney, Gabriela Kramer-Marek, Vijay Chudasama, Federico Fenaroli, Molly M. Stevens

**Affiliations:** Department of Materials, Department of Bioengineering, Institute of Biomedical Engineering, Imperial College London, London SW7 2AZ, UK; Department of Materials, Department of Bioengineering, Institute of Biomedical Engineering, Imperial College London, London SW7 2AZ, UK; Department of Materials, Department of Bioengineering, Institute of Biomedical Engineering, Imperial College London, London SW7 2AZ, UK; Department of Materials, Department of Bioengineering, Institute of Biomedical Engineering, Imperial College London, London SW7 2AZ, UK; Department of Materials, Department of Bioengineering, Institute of Biomedical Engineering, Imperial College London, London SW7 2AZ, UK; Department of Materials, Department of Bioengineering, Institute of Biomedical Engineering, Imperial College London, London SW7 2AZ, UK; Department of Materials, Department of Bioengineering, Institute of Biomedical Engineering, Imperial College London, London SW7 2AZ, UK; Department of Materials, Department of Bioengineering, Institute of Biomedical Engineering, Imperial College London, London SW7 2AZ, UK; Department of Materials, Department of Bioengineering, Institute of Biomedical Engineering, Imperial College London, London SW7 2AZ, UK; Department of Materials, Department of Bioengineering, Institute of Biomedical Engineering, Imperial College London, London SW7 2AZ, UK; Department of Materials, Department of Bioengineering, Institute of Biomedical Engineering, Imperial College London, London SW7 2AZ, UK; Department of Materials, Department of Bioengineering, Institute of Biomedical Engineering, Imperial College London, London SW7 2AZ, UK; Department of Materials, Department of Bioengineering, Institute of Biomedical Engineering, Imperial College London, London SW7 2AZ, UK; UCL Department of Chemistry, University College London, London WC1H 0AJ, UK; UCL Department of Chemistry, University College London, London WC1H 0AJ, UK; Department of Molecular Medicine, Institute of Basic Medical Sciences, Faculty of Medicine, University of Oslo, Oslo 0372, Norway; Centre for Cancer Cell Reprogramming, Institute of Clinical Medicine, Faculty of Medicine, University of Oslo, Oslo 0450, Norway; Department of Chemistry, Imperial College London, London W12 0BZ, UK; Division of Radiotherapy and Imaging, The Institute of Cancer Research, Sutton SM2 5NG, UK; LifeArc, Accelerator Building, Open Innovation Campus, Stevenage SG1 2FX, UK; Department of Life Sciences, Imperial College London, London SW7 2AZ, UK; Department of Life Sciences, Imperial College London, London SW7 2AZ, UK; Department of Chemistry, Imperial College London, London W12 0BZ, UK; Division of Radiotherapy and Imaging, The Institute of Cancer Research, Sutton SM2 5NG, UK; UCL Department of Chemistry, University College London, London WC1H 0AJ, UK; Department of Chemistry, Bioscience and Environmental Engineering, University of Stavanger, Stavanger 4021, Norway; Department of Biosciences, University of Oslo, Blindernveien 31, Oslo 0371, Norway; Department of Materials, Department of Bioengineering, Institute of Biomedical Engineering, Imperial College London, London SW7 2AZ, UK

**Keywords:** fluorescent nanoparticles, graft copolymers, polymer brushes, polymer dots, semiconducting polymer nanoparticles

## Abstract

Semiconducting polymer nanoparticles (SPNs) are explored for applications in cancer theranostics because of their high absorption coefficients, photostability, and biocompatibility. However, SPNs are susceptible to aggregation and protein fouling in physiological conditions, which can be detrimental for in vivo applications. Here, a method for achieving colloidally stable and low-fouling SPNs is described by grafting poly(ethylene glycol) (PEG) onto the backbone of the fluorescent semiconducting polymer, poly(9,9′-dioctylfluorene-5-fluoro-2,1,3-benzothiadiazole), in a simple one-step substitution reaction, postpolymerization. Further, by utilizing azide-functionalized PEG, anti-human epidermal growth factor receptor 2 (HER2) antibodies, antibody fragments, or affibodies are site-specifically “clicked” onto the SPN surface, which allows the functionalized SPNs to specifically target HER2-positive cancer cells. In vivo, the PEGylated SPNs are found to have excellent circulation efficiencies in zebrafish embryos for up to seven days postinjection. SPNs functionalized with affibodies are then shown to be able to target HER2 expressing cancer cells in a zebrafish xenograft model. The covalent PEGylated SPN system described herein shows great potential for cancer theranostics.

## 1 Introduction

Semiconducting polymer nanoparticles (SPNs), often also referred to as conjugated polymer nanoparticles or polymer dots, are an emerging class of cancer theranostics. Their utility stems from their high absorption coefficients, biocompatibility, photostability, and fluorescent brightness.^[1,2]^ SPNs are typically formed from the self-assembly of semiconducting polymers (SPs). The hydrophobic nature of the polymers makes nanoparticle formation a thermodynamically favorable process. However, this hydrophobicity also decreases colloidal stability in aqueous buffer and promotes protein adhesion, leading to protein coronas. A common way to improve colloidal stability and reduce biofouling is to coprecipitate the semiconducting polymer with amphiphilic block copolymers, lipids, or surfactants. These additives shield the hydrophobic polymers from the aqueous environment. However, the resulting binary micelles may be prone to heterogeneity, require purification, and raise questions of short- and long-term stability in a biological context.^[3]^

To circumvent the issues caused by coprecipitation, single-component systems have been explored. These commonly consist of graft copolymers of SPs with poly(ethylene glycol) (PEG) side chains. The use of these graft copolymers allows researchers a greater degree of control over the physical, chemical, and biological properties of the resulting SPNs.^[4–8]^ In addition, the optoelectronic performance of graft copolymers for cancer theranostics has been shown to be improved when compared to coprecipitation.^[9,10]^ However, graft copolymer synthesis is typically complex and potentially difficult to scale, often requiring the multistep functionalization and purification of monomer precursors and various postpolymerization modifications.^[3,8]^

In the vast majority of SPN studies for cancer targeting, accumulation of the nanoparticles at the tumor site relies on passive tumor targeting, thanks to the enhanced permeability and retention effect, which is thought to be relevant in some but not all solid tumors.^[11]^ Coating the nanoparticle surface with biorecognition elements for a cancer antigen has been shown to significantly increase tumor uptake of the nanoparticles.^[12]^ Conjugation of antibodies to SPNs has been shown by the covalent linkage of a carboxylic-acid-functionalized amphiphile to antibodies via the naturally occurring lysine residues.^[13,14]^ Typical amide-forming reactions (such as 1-ethyl-3-(3-dimethyl-aminopropyl) carbodiimide (EDC) coupling) are water and pH sensitive and as such are typically quite inefficient, requiring a large excess of both antibody and coupling agents (with respect to the nanoparticle) to achieve effective conjugation.^[13]^ Furthermore, often other stabilizing agents (e.g., PEG) and blocking proteins (e.g., bovine serum albumin) are required to prevent aggregation and improve target specificity. The modification of lysine groups is also not selective and therefore the orientation of antibodies on the SPN surface cannot be controlled. Antibody-coated SPNs have also been achieved via conjugation of streptavidin to SPNs (via EDC coupling) followed by the addition of biotinylated antibodies.^[15]^

Alternatives to antibodies for cancer nanomedicine have also been explored as a large proportion of antibodies mass is as a result of the unnecessary fragment crystallizable (Fc) region and, if the animal source is nonhuman, this region could cause immunogenicity.^[16]^ This has led to the development of nanoparticles decorated with fragment antigen binding (Fab) regions (produced via enzymatic removal of the Fc region) for cancer theranostics.^[17–19]^ For example, Fab-functionalized liposomes have been shown to have improved circulation times in vivo and as a result an improved therapeutic response when compared to the full antibody analog.^[20]^ Similar results were also observed in Fab-functionalized polymeric micelles with improved circulation efficiency and tumor penetration observed.^[21]^ Affinity proteins are another potential alternative to antibodies. They are much smaller in size (⪅20 kDa) and have comparable binding kinetics.^[22–24]^ Libraries of affinity proteins are typically generated by random mutations of specific amino acids in naturally occurring proteins via high-throughput selection technologies, such as phage display. Here, affibodies are of particular interest. They are extremely small (≈7 kDa), have excellent thermal and pH stability, and have been applied to cancer theranostic applications.^[25,26]^ Nanoparticles coated with affibodies have also been explored for cancer theranostics typically via targeting human epidermal growth factor receptor 2 (HER2) and epidermal growth factor receptor (EGFR).^[27–30]^ In particular, SPNs have also been functionalized with affibodies for theranostic purposes. Feng et al. developed SPNs for targeted dual photodynamic and photothermal therapy^[31]^ and Liu and co-workers developed SPNs for near-infrared fluorescent imaging.^[32]^ Both examples here use a coprecipitation method with a maleimide-terminated lipid to functionalize the SPNs with a cysteine-bearing affibody.

An equally important factor in nanomedicine is for the nanoparticles to circulate efficiently around the vasculature of the animal model. Zebrafish embryos have been shown to be a viable screening tool for the study of fluorescent particle circulation as they have a circulatory system and are optically transparent.^[33]^ Xenograft models of zebrafish can also be generated with relative ease. This allows for the study of nanoparticle–cancer cell interaction with a level of detail that would be very difficult to achieve with mouse models.^[34]^ SPNs have been explored in zebrafish for the study of particle accumulation,^[35]^ glutathione sensing,^[36]^ brain imaging,^[37]^ and biocompatibility.^[34,35,38]^ In addition, SPNs coated with hyaluronic acid were shown to inhibit cluster of differentation-44 (CD44) expressing tumor growth in zebrafish xenograft models.^[39]^

In this work, we employ a one-pot, postpolymerization nucleophilic aromatic substitution reaction (S_N_Ar) to covalently attach PEG side chains onto the backbones of SPs containing a fluorinated benzothiadiazole (BT) unit.^[40,41]^ Fluorinated BTs are commonly found in a variety of donor–acceptor semiconducting polymers.^[42]^ Therefore, methodologies that employ modifications of this unit, postpolymerization, are favorable due to the lack of bespoke monomer synthesis needed.^[43]^ We use the fluorescent poly(9,9-dioctylfluorene-*alt*-benzothiadiazole) (F8BT) as a proof-of-concept semiconducting polymer. When the PEGylated F8BT graft copolymers are precipitated into water, the resulting SPNs exhibit excellent colloidal stability in a range of buffer solutions and low protein fouling in biological media (fetal bovine serum, FBS) without the need for any surfactants. The modular nature of this reaction also allows for the inclusion of azide-terminated PEG side chains which yields SPNs with a surface capable of modification via the efficient strain-promoted azide–alkyne cycloaddition (SPAAC) reaction. Here, we show an example of this modification by affixing, onto the surface of the nanoparticles, antigen-targeted proteins (antibody, Fab, and affibodies) bearing site-specific strained-alkyne functionality. These targeted nanoparticles all exhibited the ability to bind to cancer cells expressing the target antigen HER2, with high specificity. The circulation efficiency of the PEGylated SPNs was studied in zebrafish embryos and were observed to keep circulating for at least seven days postinjection. Finally, affibody-coated SPNs were microinjected into xenograft zebrafish models and were shown to have a significant improvement in cell association when compared to the nontargeted control.

## 2 Results and Discussion

We designed and tested PEG graft copolymers to achieve single-component SPNs with good colloidal stability, reduced biofouling in physiological environment, bright fluorescence, and tunable surface functionality. All are key considerations for downstream applications in cancer imaging.

### 2.1 Grafted PEG Length Can Be Tuned to Maximize Colloidal Stability

**Figure 1:**
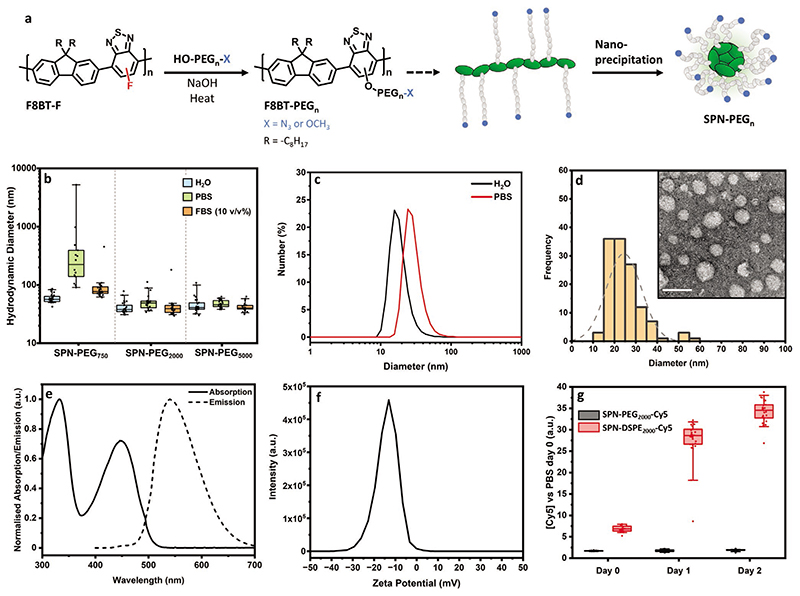
Synthesis and characterization of SPNs. a) Scheme of F8BT-F modification via S_N_Ar reaction with poly(ethylene glycol) derivatives and subsequent nanoparticle formation. b) Hydrodynamic diameter of SPNs in water, PBS (at room temperature), and 10 v/v% FBS (in PBS, at 37 °C) after 24 h of incubation, calculated from FCS autocorrelation analysis. c) Hydrodynamic diameter of SPN–PEG_2000_ in water and PBS after 24 h of incubation, via DLS analysis (average of *n* = 3 technical replicates). d) TEM image and the corresponding histogram of SPN–PEG_2000_ (*n* = 126 particles counted, mean diameter of 24 ± 8 nm, scale bar = 50 nm). e) Normalized absorption and fluorescence spectra (*λ*_Ex_ = 450 nm) of SPN–PEG_2000_ in water. f) Zeta potential of SPN–PEG_2000_ in water (average of *n =* 3 technical replicates). g) FCS analysis of coprecipitated (with DSPE–PEG_2000_) and graft copolymer SPNs (with Cy5-labeled PEG chains) incubated in FBS. Reported values are the concentration changes in the red channel (Cy5 fluorescence). All FCS data above is *N* = 1, *n* ≥ 14 technical replicates. Box plots: 5th and 95th percentile values (top and bottom horizontal lines), the lower quartile (lower boundary of the box), the median (the line inside the box), and the upper quartile (upper boundary of the box).

We first investigated the length of the grafted PEG that was required to achieve colloidal stability. The monofluorinated derivative of F8BT (poly(9,9′-dioctylfluorene-5-fluoro-2,1,3-benzothiadiazole) (F8BT-F, Figure 1a) was synthesized by Suzuki polymerization, based on our previously reported method.^[41]^ We then chose three different molecular weights (750, 2000, and 5000 Da) of poly(ethylene glycol) methyl ether (HO–PEG_*n*_–OMe) to graft onto the backbone of F8BT-F in a one-step nucleophilic aromatic substitution reaction (Figure 1a).^[40,41]^ The resulting graft copolymers were dispersed as aqueous nanoparticles via nanoprecipitation, in which the polymer solution (in tetrahydrofuran (THF)) is injected rapidly into water (followed by evaporation of the THF). The respective dispersions are referred to as SPN–PEG_750_, SPN–PEG_2000_, and SPN–PEG_5000_.

To test the stability of the PEGylated SPNs in physiologically relevant solutions, the nanoparticles were then diluted into water, phosphate buffered saline (PBS) (at room temperature), and PBS with 10 v/v% FBS (at 37 °C) and monitored for 24 h. The resulting dispersions were analyzed by fluorescence correlation spectroscopy (FCS), using the inherent fluorescent signal from SPNs to assess the colloidal stability over time (see Figures S1–S3 in the Supporting Information for FCS at all time points for SPN–PEG_750_, SPN–PEG_2000_, and SPN–PEG_5000_, respectively). Figure 1b shows the collated summary of the SPNs after 24 h incubation. SPN–PEG_750_ exhibited immediate signs of aggregation in PBS, characterized by an increase in hydrodynamic diameter, but showed good stability in the presence of FBS. SPN–PEG_2000_ and SPN–PEG_5000_ showed no evidence of any significant size change in both solutions, which indicates high colloidal stability at physiological temperature and in the presence of serum proteins. Dynamic light scattering (DLS) of SPNs in PBS also showed the same trend. The DLS number distribution of SPN–PEG_2000_ in water and PBS can be found in Figure 1c. See Figures S4–S6 in the Supporting Information for the summary of all DLS number, volume, and intensity distributions, respectively. Due to the scattering of proteins, DLS could not be performed on any samples containing FBS.

To ensure that covalent grafting of the PEG was required for colloidal stability, particles were also fabricated by simply mixing the appropriate ratio of HO–PEG_*n*_–OMe and F8BT together in THF, followed by nanoprecipitation. These particles all showed instability after 8 h in PBS by FCS measurements (Figure S7, Supporting Information). This confirms that grafting PEG to the F8BT backbone is key to impart colloidal stability.

Although SPN–PEG_750_ was serum stable in presence of FBS, its instability in PBS alone indicates that protein fouling on the surface is essential to provide stability. In addition, SPN–PEG_5000_ was more challenging to purify due to its partial solubility in water. Hence, SPN–PEG_2000_ was investigated in more detail for the rest of this work. Under transmission electron microscopy (TEM), with negative staining, SPN–PEG_2000_ particles had an average diameter of 24 ± 8 nm (Figure 1d). This compares well to the DLS number distribution (Figure 1c) taking into consideration the hydration shell that is included in DLS but not in TEM. The majority of characterization in this study will focus on the nanoparticles in their hydrated form as that is more relevant for the desired application. Normalized absorption and emission spectra of SPN–PEG_2000_ are shown in Figure 1e. When compared to F8BT-F, the spectra exhibit a lowering of the absorption band at 450 nm (relative to the band at 330 nm) and small redshift in fluorescence upon inclusion of the PEG, which has been observed with previous S_N_Ar modifications of F8BT-F (see Figure S8 in the Supporting Information for overlayed spectra).^[41]^ The zeta potential was found to peak at ≈–10 mV (Figure 1f).

Overall, we synthesized graft copolymers with an increasing length of PEG side chains and found that a molecular weight of 2000 Da was sufficient to achieve SPNs with good colloidal stability in PBS and in the presence of serum proteins, at physiological temperature.

### 2.2 Graft Copolymer Approach Yields Nanoparticles More Stable than Coprecipitated Nanoparticles

An alternative way of generating SPNs with improved colloidal stability is to mix an unmodified semiconducting polymer with an excess of a biocompatible amphiphilic material (such as a surfactant, lipid, or block copolymer) prior to nanoprecipitation.^[1,44]^ This has been widely employed in the development of SPNs as a wide range of SPs can simply be mixed with these commercially available materials. To compare these noncovalent methods with our covalent system, we have also included PEGylated lipids in our study, due to the prevalence of their use as surfactants in the literature and the ease of obtaining azide-functionalized derivatives for labeling of the PEG in subsequent stability experiments.^[45–50]^

Lipid–SPNs were generated by mixing F8BT-F with an excess of a common PEG lipid (1,2-distearoyl-sn-glycero-3-phosphoethanolamine-N-[methoxy(polyethylene glycol)-2000] (18:0 DSPE–PEG_2000_ PE)) followed by removal of excess lipid via centrifuge filtration. The resulting nanoparticles (labeled SPN–DSPE_2000_) showed very similar stability performance in PBS and PBS with 10 v/v% FBS (at 37 °C) to that of SPN–PEG_2000_ (Figure S9, Supporting Information).

We hypothesized that, although the addition of these lipids improved the colloidal stability dramatically, the reversible nature of the noncovalent interactions associating the surfactant with the polymer could lead to lipid dissociating from the surface, forming surfactant micelles. In order to investigate this, azide-functionalized SPNs were fabricated by both the grafted and lipid mixing methods. The azide-terminated graft copolymer was synthesized by reacting F8BT-F with HO–PEG_2000_–N_3_ (instead of the methyl ether analog). Analogous lipid particles were assembled by coprecipitation of F8BT-F with 18:0 DSPE–PEG_2000_–N_3_. Both nanoparticle suspensions were reacted with a large excess of dibenzocy-clooctyne–cyanine-5 (DBCO-Cy5) overnight. The excess dye was then removed via centrifuge filtration. The resulting dye-labeled particles (SPN–PEG_2000_–Cy5 and SPN–DSPE_2000_–Cy5, respectively), enabled the independent analysis of the Brownian motion of the semiconducting polymer (in the green channel) and the Cy5-labeled PEG (in the red channel) by two-color FCS analysis. Cross-correlation analysis was not possible due to the broad fluorescence spectrum of F8BT particles, which causes some crosstalk between the channels.

Dye-labeled particles were incubated in FBS for 48 h (at 37 °C) and the concentration of individual diffusing components in both channels was recorded at regular timepoints by two-color FCS (all concentrations were compared to their initial measurement in PBS). SPN–PEG_2000_-cy5 exhibited no significant change in the red or green channel indicating that the number of diffusing species remained similar over time, confirming high colloidal stability of the covalent system. By contrast, the lipid two-component system showed a large increase in the relative concentration of diffusing species in the red channel over time (Figure 1f), while in the green channel the concentration remained the same (Figure S10a, Supporting Information). This demonstrates that the two-component system is disintegrating over time, with DSPE_2000_–Cy5 partitioning out of the comixtures to form separate particles. Both SPNs, however, exhibited no significant change in PBS over the same time period in the red and green channels (Figure S10b,c, respectively, Supporting Information). This suggests that the lipids dissociated from the blend particle surface in the presence of protein-rich environments, forming more particles, whereas this did not occur when the PEG was covalently bound to the SP in our SPN–PEG_2000_. If this model dye (Cy5) is replaced with a targeting moiety such as an antibody, for example, this would be an issue for targeted bioimaging and therapeutic applications, when using the blended system. This observation demonstrates a clear advantage of our covalent versus a noncovalent blend system for subsequent biomedical applications.

### 2.3 PEG Grafting Density Can Be Tuned to Maximize Colloidal Stability and Minimize Protein Corona Formation

**Figure 2:**
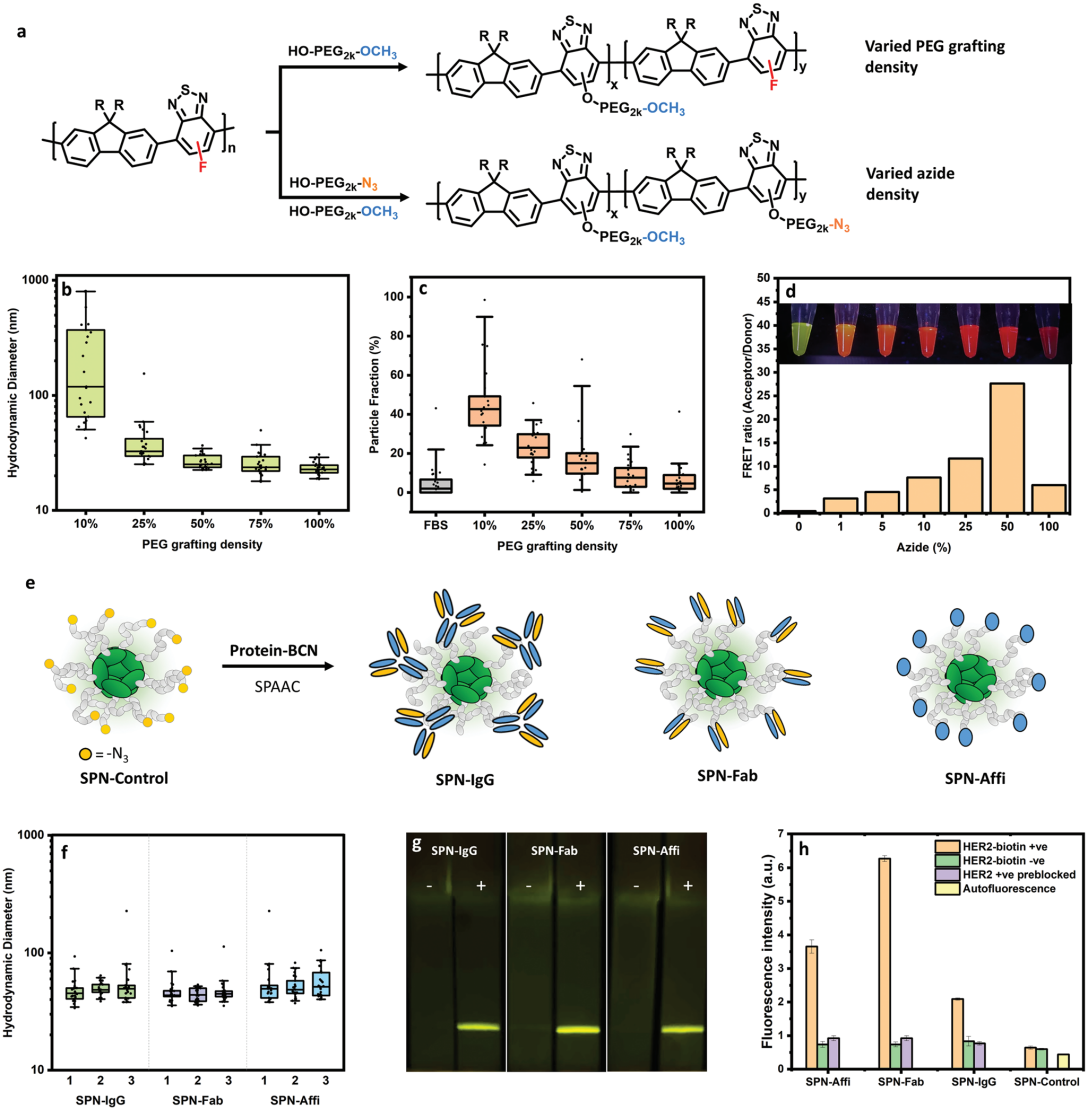
Synthesis and characterization of SPNs with tuned grafting density, azide density, and cancer targeted proteins. a) Scheme of modification of F8BT-F with varied PEG grafting and azide densities. b) Hydrodynamic diameter of SPNs with a varying PEG grafting density in PBS after 24 h incubation calculated from FCS autocorrelation analysis (*n* = 25 technical replicates). c) Particle fractions from two-component fits of FCS curves after mixing SPN series with FBS–AF647, revealing extent of protein fouling on nanoparticle surface, with high particle fraction corresponding to high protein binding (*n* = 25 technical replicates). d) Ratio of acceptor emission (*λ*_Em_ = 630 nm) and donor emission (*λ*_Em_ = 540 nm) of AF594-functionalized particles, as a function of azide-functionalization percentage. Image insets show SPN solution under UV-light exposure (*n* = 1). e) Scheme for the modification of SPN–PEG_2000_–N_3_ with byclonononyne (BCN)-functionalized IgG, Fab, and affibody. f) Hydrodynamic diameter of SPNs in PBST (PBS with 0.05 v/v% Tween-20) calculated from FCS autocorrelation analysis (3 independent particle batches, *n* = 25 technical replicates for each). g) Images of lateral flow strips (with a polystreptavidin test line) which have been wicked with SPNs with (+) and without (–) HER2 biotin (imaged under blue light (450 nm) with a SYBR-gold filter). h) FLISA fluorescence intensity data (*λ*_Ex_ = 450 nm, *λ*_Em_ = 540 nm) of SPNs with and without HER2 biotin, including a blocked HER2 biotin control (data shown as mean ± S.D., *N* = 1, *n* = 3 technical replicates. Box plots: 5th and 95th percentile values (top and bottom horizontal lines), the lower quartile (lower boundary of the box), the median (the line inside the box), and the upper quartile (upper boundary of the box).

Tuning of the PEG grafting density was explored as it was suspected that affixing a PEG chain on every repeat unit was not essential to achieving good colloidal stability and reduce protein fouling. F8BT-F was reacted with decreasing equivalents of HO–PEG_2000-_–OMe (100, 75, 50, 25, and 10 mol%), yielding a series of graft copolymers (Figure 2a). NMR analysis was used to calculate amount of PEG grafted onto F8BT-F for each polymer in the series. In each case, the reaction was found to be quantitative, for example, reacting 25 mol% of PEG resulted in a polymer with a 25% grafting density (see the Supporting Methods in the Supporting Information). UV–vis of the polymer series exhibited the similar relative drop in the absorption band at 450 nm compared to the band at 330 nm discussed previously (Figure S11, Supporting Information).

As before, SPN solutions were prepared via the nanoprecipitation method from polymers with a varied grafting density. Stability was studied via FCS and DLS as before. No aggregation in PBS was observed under FCS analysis after 24 h, except for the 10 mol% graft copolymer (Figure 2b, earlier time points of FCS data can be found in Figure S12 in the Supporting Information). No aggregation was observed in water and in the presence of FBS after 24 h for the graft series (Figures S13 and S14, Supporting Information, respectively). Long-term stability in PBS was also assessed by DLS and again, only aggregation was observed in the polymer with 10 mol% PEG grafted (Figure S15, Supporting Information). We hypothesize that the reduction in colloidal stability of the 10 mol% polymer is due to the insufficient proportion of hydrophilic PEG to the hydrophobic semiconducting polymer backbone. Zeta potential was also recorded on the series and no trend with grafting density was observed, with all particles exhibiting a mean zeta potential of less than –10 mV (Figure S16, Supporting Information). This reveals that a minimum of 25 mol% PEGylation of F8BT-F is required to deliver colloidally stable nanoparticles in PBS in the absence of any other stabilizing agents such as serum proteins.

The protein fouling behavior (protein corona formation) was also investigated for this series as low protein fouling has been shown to lead to improved circulation efficiencies in vivo.^[51]^ SPNs were incubated with a variant of FBS in which the proteins had been tagged with AlexaFluor647 (via random lysine conjugation). By observing the Brownian motion of the dye-labeled serum components (red channel), it was determined in situ whether the proteins are diffusing freely in solution or are bound to a nanoparticle surface.^[52,53]^ This analysis provides a direct in situ measure of protein corona formation. Figure 2c shows the particle fractions obtained from this two-component FCS analysis (red channel), with higher particle fractions corresponding to higher protein binding. There is a clear trend of decreased protein fouling with increasing PEG density, as expected. This also confirmed the above hypothesis that particles with 10 mol% PEG bind high amounts of protein that helped to stabilize the particles as shown when looking at the particle size in presence of serum (Figure S14, Supporting Information). Ultimately, this shows that although a minimum of 25 mol% PEGylation was required for colloidal stability, 75–100 mol% is required to minimize all protein interactions.

### 2.4 Proportion of Reactive Groups on the Nanoparticle Can Be Controlled

As we showed above, reactive azide groups can be easily added by swapping the methyl ether PEG for the azide-terminated analog (HO–PEG_2000_–N_3_) used in the synthesis. The azide groups allow for the functionalization of the nanoparticle surface with a wide array of targeting biomolecules such as proteins, nucleic acids, and peptides, which are often provided with strained-alkyne functionality from commercial suppliers. This simplifies the way in which a wide array of functional SPNs can be fabricated from one common material.

We first investigated whether the proportion of azide groups on the nanoparticle surface can also be carefully controlled. Graft copolymers were synthesized with different ratios of azide to methyl-ether-terminated PEG alcohol (Figure 2a). The proportion of azide groups was varied from 1, 5, 10, 25, 50 to 100 mol% (see the Supporting Information for UV–vis (Figure S17, Supporting Information)) and NMR characterization of the series (Supporting Methods, Supporting Information)). The resulting SPNs were then reacted with an excess of AlexaFluor594 (DBCO–AF594), a suitable Förster resonance energy transfer (FRET) acceptor, and purified. Fluorescent spectra were recorded by exciting at the absorption maxima of the semiconducting polymer (FRET donor). The resulting spectra (Figure S18, Supporting Information) and extracted acceptor to donor ratio (emission maxima of acceptor/donor, Figure 2d) clearly show that increasing the proportion of azide groups results in more dyes attached to the particle at close proximity to the semiconducting polymer (i.e. on the nanoparticle surface), as indicated by an increase in the acceptor–donor ratio. This is also apparent by the color of the suspensions under UV exposure (Figure 2d). Interestingly, the SPN with 100 mol% azide groups resulted in a lower ratio. This is most likely due to aggregation-induced quenching as a result of the abundance of dye on the nanoparticle surface. Overall, we have shown here that the proportion of reactive azides on the surface of the SPN can be carefully controlled. Therefore, the amount of the strained-alkyne-functionalized molecule (using DBCO–AF594 here as an example) which reacts with the surface is also tunable.

### 2.5 Azide Functionality Allows for Nanoparticles to be Conjugated to Anti-HER2 Proteins

The azide functionality allows for SPNs to be decorated with alkyne-containing biomolecules via the SPAAC reaction. Here, we chose to tailor our SPNs for cancer targeting by affixing biorecognition elements to the surface.

For this study, an azide-functionalized polymer was synthesized as before using 50 mol% PEG density (this PEG grafting density was chosen here as a trade-off between colloidal stability (Figure 2a) and the economical use of the more expensive PEG–azide derivative). Up to this point, the focus on colloidal stability has been at physiological pH. However, common bioconjugations require a wider pH range so it was important to test the colloidal stability of the azide-functionalized SPNs in a variety of buffer solutions (pH 5.4–9.8) at their typical buffer strengths (50–100 mm). The hydrodynamic radius and per-particle brightness were investigated after four days in solutions at room temperature via FCS. SPNs exhibited no aggregation and a preserved high particle brightness (≈20–30-fold brighter than AlexaFluor488 using 488 nm excitation) in all buffers (Figure S19, Supporting Information). TEM images of these particles (with negative staining) showed particles with an average diameter of 16.5 ± 4.9 nm (Figure S20, Supporting Information).

In order to target cancer cells, we chose three different biomolecules to functionalize on the nanoparticle surface (via the SPAAC reactions): an antibody (immunoglobulin G (IgG), Ontruzant), its antigen-binding antibody fragment (Fab, Ontruzant), and an affibody (Z_HER2:2395_–Cys) which all target the common cancer associated protein HER2 (ERBB2). Strained alkynes were installed onto the proteins using bromo/dibromopyridazinedione chemistries. The monobromo derivative was selective for cysteine residues (Z_HER2:2395_–Cys) and the dibromo for disulfide bridges (Ontruzant, Ontruzant Fab).^[54]^ See Figure S21 in the Supporting Information for chemical structures of both pyridazinedione linkers. Previous work by Bahou and Chudasama have shown that these linkers have negligible impact on stability and antigen binding.^[55]^ The reaction yielded IgG, Fab, and affibody with at least one strained alkyne at specific sites of each protein. The resulting byclonononyne (BCN)-functionalized proteins were conjugated to SPN–PEG_2000_–N_3_ and purified from free protein to create SPN–IgG, SPN–Fab, and SPN–Affi (Figure 2e). Particles were synthesized in triplicate and exhibited no signs of aggregation and a very similar hydrodynamic diameter between batches (Figure 2f).

The binding of the biomolecule-functionalized SPNs to HER2 was then assessed in both a lateral flow immunoassay (LFIA) and fluorescence-linked immunosorbent assay (FLISA) formats. LFIAs consisted of cellulose paper strips with a printed polystreptavidin line. SPNs were wicked up the strips with and without preincubation with HER2 biotin (Figure 2g). Fluorescence at the test line was only observed in the presence of biotinylated HER2; this provides clear evidence of HER2 targeting ligands on the surface of the SPNs. These results were consistent with the result of FLISA experiments, in which, streptavidin-coated microwell plates were incubated with and without HER2–biotin, followed by SPN incubation (unfunctionalized SPNs are referred to as SPN-Control herein). An additional control experiment was also performed in this case (to rule out nonspecific binding), where the HER2–biotin plates were preblocked with unconjugated anti-HER2 (IgG or affibody) before SPN incubation (Figure 2h). In all cases, there was a large contrast between the HER2 positive sample and the controls, confirming that the SPNs had the ability to bind specifically to the HER2 protein. Furthermore, the controls exhibited very little signal which suggests that SPNs display promising antifouling properties.

### 2.6 Nanoparticles Can Bind to HER2 In Vitro with High Photostability

**Figure 3:**
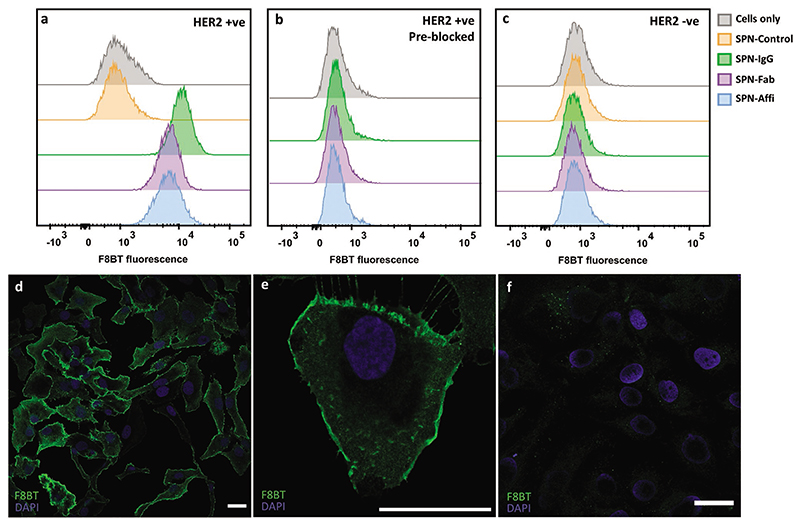
Targeted SPNs in vitro. Representative histograms from flow cytometry of a) SKOV3 cells (HER2 positive), b) SKOV3 cells (preblocked with free IgG/affibody) incubated with targeting and control SPNs, c) MDA-MB-468 cells (HER2 negative) incubated with SPNs. d,e) Confocal images of SKOV3 cells incubated with SPN–Affi. f) Confocal images of SKOV3 cells incubated with SPN-Control. Scale bars = 30 μm.

The ability of SPN–IgG, SPN–Fab, and SPN–Affi to target HER2 expressing cells was then studied. Functionalized SPNs (1 nM) were incubated with SKOV3 cells (an ovarian cancer cell line which overexpresses HER2) at 4 °C to prevent any SPN internalization. Cells were then analyzed by flow cytometry to evaluate the targeting efficiency of each system. Representative histograms can be seen in Figure 3a and all extracted cell association values (obtained by gating the positive population based on the “cell only” control) can be found in Figure S22 (Supporting Information). Representative side scatter (SSC) versus forward scatter (FSC) dot plots can also be found in Figure S23 (Supporting Information). In all cases, a greater than 95% cell association was found for each targeted SPN. Blocking the HER2 with a large excess of free anti-HER2 IgG or affibody reduced the cell association to less than 0.3% (Figure 3b), confirming the specificity of the SPNs for HER2 (SPN-Control was not considered for this study). To further confirm the SPN–cell interaction is HER2 specific, the HER2 negative MDA-MB-468 cell line was incubated with SPNs under the same conditions. This also resulted in less than 0.3% cell association, as seen by the absence of F8BT fluorescent signal. This, in conjunction with the LFIA and FLISA data, indicates that the association of SPNs is via specific binding to the HER2 protein, with excellent specificity.

The cytocompatibility of the SPNs was investigated by incubating an increasing concentration of SPNs (from 0.25 to 20 nm) with the epithelial cell line HEK-293 for 72 h (Figure S24, Supporting Information). For the functionalized SPNs, cytotoxicity was only observed for the highest concentrations (10–20 nm) which was beyond the working concentration used in the above study (1 nm). A more in-depth cytocompatibility evaluation with other cell types will be interesting in the future.

To visualize that functionalized SPNs were binding to HER2 receptor, we performed confocal imaging. For this purpose, SPN–Affi was chosen and compared to the unfunctionalized particles. In all cases, SKOV3 cells were incubated with SPNs at 4 °C to prevent nonspecific cell internalization. Representative images showed strong binding of SPN–Affi to the cell membrane, while no green fluorescence was detected for the Control-SPN confirming the specific labeling of HER2 (Figure 3d–f). 3D confocal reconstructions of SPN–Affi support this and show fluorescent signal only on the membrane of the cells, where the HER2 protein is located (Video S1, Supporting Information). A further representative confocal image, which includes a brightfield channel, can be found in Figure S25 (Supporting Information).

The photostability of SPN–Affi was investigated in line with a dye-based analog (IgG conjugated to AlexaFluor-488 (IgG–AF488). IgG–AF488 was used to stain the SKOV3 cells as above. To accelerate photobleaching, cells were then illuminated at the maximum laser power within a 40× objective window every 10 s for 30 min. The photobleaching half-life was found to be ≈7–8 min for IgG–AF488 and 15 min for Affi–SPNs (Figure S26, Supporting Information). The SPNs could therefore be imaged for nearly twice as long as the dye-conjugated IgG before reaching the same level of relative photobleaching. Images of the same area at a 20× magnification were then taken to show the extent of photobleaching (Figure S27, Supporting Information) and time-lapse videos of the photobleaching process can be found in Video S2 (Supporting Information). This showcases that targeting SPNs are suitable for confocal imaging and are more photostable than conventional dyes.

### 2.7 PEG-Grafting Nanoparticles Have High Circulation Efficiency In Vivo

In order for the SPNs to be effective for use in vivo, the particles need to have a good circulation efficiency to minimize nonspecific tissue retention and maximize target binding. Zebrafish embryos were chosen as they are an ideal model for fluorescent nanoparticle imaging due to their optical transparency. Work by Dal et al. has shown that the circulation efficiency in zebrafish closely correlates to that of mice.^[33]^

**Figure 4:**
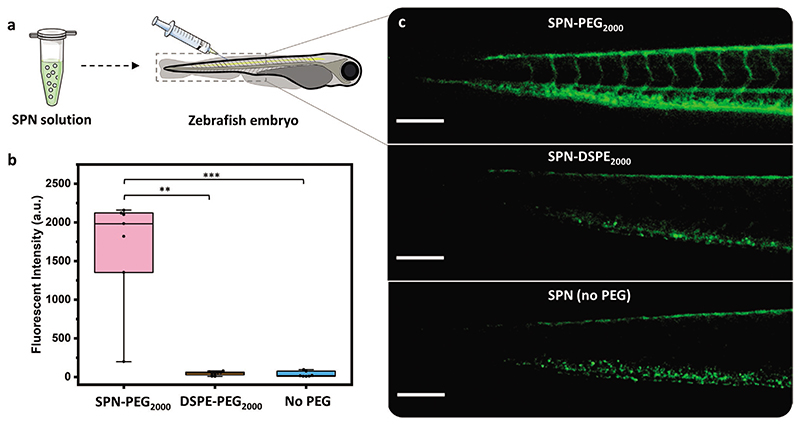
Circulation of SPNs in zebrafish embryos. a) Scheme of SPN injection into the caudal vein of zebrafish embryos. b) Fluorescence intensity of the circulating SPNs extracted from video analysis 8 h postinjection. *n* ≥ 7 embryos per group, statistical analysis: Kruskal–Wallis test with post-hoc Dunn’s test, comparison to SPN–PEG_2000_, **p < 0.01 and ***p < 0.001. c) Representative stereomicroscope fluorescence images of the tail region of zebrafish embryos, 8 h postinjection (scale bar = 200 μm). Box plots: 5th and 95th percentile values (top and bottom horizontal lines), the lower quartile (lower boundary of the box), the median (the line inside the box), and the upper quartile (upper boundary of the box). Figure a) was partly generated using Servier Medical Art, provided by Servier, licensed under a Creative Commons Attribution 3.0 unported license.

Embryos were microinjected in the caudal vein (CV) with suspensions of SPN–PEG_2000_ and SPN–DSPE_2000_ in PBS. SPNs consisting of bare F8BT-F (with no PEG) were also included (in pure water as the particles were unstable in PBS) (Figure 4a). Images and videos of each embryo at set time points (8, 24, and 48 h postinjection) were recorded using a stereomicroscope. Qualitatively, in the representative videos it can be clearly seen that SPN–PEG_2000_ is circulating in the zebrafish vasculature throughout the 48 h (see Videos S3–S5 in the Supporting Information for 8, 24, and 48 h postinjection videos, respectively). However, in the case of lipid-coated (SPN–DSPE_2000_) and bare particles (SPN no PEG), videos show no fluorescence in the lumen of the vessels after only 8 h postinjection. This is also reflected in the image also taken 8 h postinjection, where PEG-grafted particles are distributed around the entire embyro vasculature but the lipid and bare particles have been removed from the vasculature and had formed clusters (Figure 4c). This is most likely due to uptake of lipid-coated and bare SPNs into the endothelial and/or macrophage cells, which remove them from circulation.^[33]^ Whereas this occurs to a lower extent for the PEG-grafted nanoparticles (SPN–PEG_2000_). Representative images of each SPN series at 24 and 48 h time points can be found in Figures S28 and S29 (Supporting Information), respectively.

A more quantitative assessment was performed to evaluate the relative intensity of the circulating particles from the videos recorded 8 h postinjection (see the Supporting Methods in the Supporting Information for details of video processing). The resulting fluorescent intensity plots (Figure 4b) clearly show that the SPN–PEG_2000_ has a significantly improved circulation efficiency compared to lipids and bare SPNs. The relatively poor performance of SPN–DSPE_2000_ can possibly be explained by the findings discussed above where we observed the lipids to be labile in protein rich environments (Figure 1e,f).

As a result of the excellent performance of SPN–PEG_2000_, further embryos were injected with this SPN alone and the fishes were monitored over longer time points. SPN–PEG_2000_ was still observed to be circulating after seven days postinjection (Video S6, Supporting Information). A further high-resolution video of SPN–PEG_2000_ circulating through the artery and around the heart, 24 h postinjection can be found in Video S7 (Supporting Information). The negligible protein corona observed with SPN–PEG_2000_ (Figure 2c) could partly explain the high circulation observed, as this correlation has been shown in work with other nanoparticle systems.^[51]^ To the best of our knowledge, this is the first example of such long circulating SPNs and a rare example of any other nanoparticle circulating for such an extended period of time.^[51]^

### 2.8 Affibody-Functionalized Nanoparticles Can Target HER2 In Vivo

**Figure 5:**
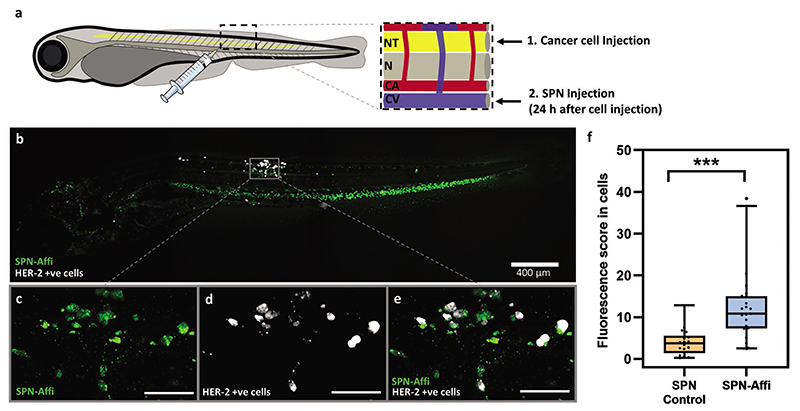
Targeted SPNs in zebrafish xenografts. a) Schematic representation of cancer cell (pretreated with CellMask deep red stain) and SPN injection (24 h after cancer cell injection) into the zebrafish embryo neural tube (NT) and caudal vein (CV), respectively. “N” and “CA” refer to the natural tube and the caudal artery, respectively. b) Representative confocal image of a zebrafish embryo xenograft 24 h after SPN–Affi injection. c–e) The solid box area is enlarged, showing the fluorescent signal in the confocal slice for SPN–Affi, cancer cells, and SPN–Affi/cancer cells combined, respectively (scale bar = 100 μm). f) Quantification of the average fluorescence intensity from 3D reconstruction images of SPN–Affi and SPN-Control inside cancer cells (*n* ≥ 18 embryos per group). Statistical analysis: Kolmogorov–Smirnov test, comparison to SPN-Control, ***p < 0.001. Box plots: 5th and 95th percentile values (top and bottom horizontal lines), the lower quartile (lower boundary of the box), the median (the line inside the box), and the upper quartile (upper boundary of the box). Figure a) was partly generated using Servier Medical Art, provided by Servier, licensed under a Creative Commons Attribution 3.0 unported license.

SPN–Affi was explored further for cancer targeting in vivo as affibody-coated nanoparticles for cancer theranostics are relatively unexplored. This was assessed by using HER2 positive zebrafish embryo xenografts. This model was generated by injecting HER2-expressing SKBR-3 cells (preincubated with deep red CellMask labeling dye) into the neural tube (NT) and allowing them to grow for 24 h (Figure 5a). SPNs (SPN–Affi and SPN-Control) were then injected intravenously and the zebrafish xenografts imaged, under confocal microscopy, after a further 24 h. The high transparency of the zebrafish models allowed the quantification of the cellular uptake of SPNs in vivo. The representative confocal images of SPN–Affi can be seen in Figure 5b–e, where the association of SPN–Affi with cancer cells can be clearly observed. Whereas, for the SPN-Control, much less association can be seen (Figure S30, Supporting Information). Also, of note in the representative image (Figure 5b) is the large fluorescent band observed in the tail region of the fish. This is most likely particles being uptaken into macrophage and endothelial cells along the caudal vein and is commonly observed with long circulating particles.^[33,56,57]^ The resulting 3D images of each fish were analyzed for the SPN fluorescence signal only found within the cancer cells. This gave a fluorescence score for each embryo. The resulting compiled fluorescent scores showed a clear significant difference of SPN–Affi versus the control nanoparticles (Figure 5f, see the Supporting Methods in the Supporting Information for details of the image analysis). This confirms that affixing the affibody to the surface of the SPNs increases the cell association in vivo when compared to the nontargeting control.

## 3 Conclusion

By grafting azide-terminated poly(ethylene glycol) onto the backbone of semiconducting polymers, we can formulate fluorescent semiconducting polymer nanoparticles which have excellent colloidal stability, cancer targeting capability, and circulation efficiency in zebrafish embryos, rivaling that of the best performing nanoparticles reported in the literature. The advantage of the approach described here is that the particles are inherently fluorescent so, unlike other long circulating nanoparticles (e.g., polymeric micelles and liposomes), do not require loading or functionalization with a fluorophore.^[58,59]^

This work was designed to showcase a modular way in which this postpolymerization methodology can be used to tailor SPNs for the desired application. Taking our in vivo targeting of zebrafish embryos as an example; further optimization of the PEG grafting and azide density could be performed to find that optimum balance of size, circulation efficiency, and tumor targeting, to ultimately achieve a much greater labeling efficiency. In addition, the azide-decorated SPNs could be applied to a variety of other applications in nanoparticle-based diagnostics and therapeutics, by simply switching out the alkyne-functionalized protein with other biomolecules (e.g., nucleic acids, peptides, sugars, etc.). Finally, for further in vivo applications in mouse models and beyond, it is often desirable for the SPN to absorb in the near-infrared window for deeper tissue penetrations. This could be achieved by switching the fluorene (F8) comonomer in F8BT-F to a more electron deficient monomer in order to lower the bandgap and shift the absorption into this window.

## 4 Experimental Section

Further details of equipment and reagents can be found in the Supporting Methods (Supporting Information).

### Polymer Synthesis

See the Supporting Methods in the Supporting Information for all details of polymer synthesis.

### PEG-Grafted SPN Assembly (Nanoprecipitation)

Graft copolymer dissolved in THF (100 μL, 1 mg mL^–1^) was filtered (0.45 μm, polytetrafluoroethylene (PTFE)) and injected rapidly into milli-Q water (1000 μL). The resulting solution was left open for several hours to allow THF to fully evaporate, yielding nanoparticle solutions.

### Lipid-Coated SPN Assembly (Nanoprecipitation)

F8BT-F (1 mg mL^–1^) and lipid (18:0 DSPE–PEG_2000_ PE or 18:0 DSPE–PEG_2000_–azide) dissolved in THF was filtered (0.45 μm, PTFE) and injected rapidly into milli-Q water (100 μL into 1000 μL). The resulting solution was left for several hours to allow THF to evaporate, yielding nanoparticle solutions. Solutions were purified by centrifuge filtration (Amicon, 100 kDa molecular weight cut-off (MWCO)) 5 times with milli-Q water to remove excess lipid.

### FCS Measurements

5 μL sample was placed in an ibidi 8-well plate (80827, ibidi, Germany) inside the incubation chamber (37 °C) of a commercial LSM 880 (Carl Zeiss, Jena, Germany). A 40× C-Apochromat water immersion objective (numerical aperture NA 1.2) was used to focus the laser beams 200 μm above the bottom glass plate. Ar^+^ laser (488 nm), HeNe lasers (561 and 633 nm), and appropriate filter sets were used for all FCS measurements. All data were fitted using PyCorrfit program 1.1.6.^[60]^ Measurements to calibrate the beam waist $\left(\omega_{\text{xy}}^{2}\right)$ to obtain diffusion coefficients (*D*) from diffusion times (*τ_D_*) for all the subsequent unknown samples were first conducted on free OG488 in PBS (*D* = 5.49 × 10^–6^ cm^2^ s^–1^ at 37 °C, *D* = 4.1 × 10^–6^ cm^2^ s^–1^ at 25 °C), sulforhodamine B in PBS (*D* = 5.54 × 10^–6^ cm^2^ s^–1^ at 37 °C, *D* = 4.14 × 10^–6^ cm^2^ s^–1^ at 25 °C), and Alexa647 in PBS (*D* = 4.42 × 10^–6^ cm^2^ s^–1^ at 37 °C, *D* = 3.3 × 10^–6^ cm^2^ s^–1^ at 25 °C).^[61]^ *D* was converted to hydrodynamic diameters using Stokes–Einstein equation. For each sample, 25–30 intensity traces of 5 s each were recorded and autocorrelated. Most data, except the FBS–AF647 series, were analyzed with one component fits *G*_1comp_(*τ*) to yield diffusion times *τ_D_* and the number of particles in the confocal volume (*N*). A triplet fraction *T* with the corresponding triplet time *τ*_trip_ was added and fixed between 1 and 10 μs. The structural parameter SP was always kept constant at a value of 5. Curves below the limit of detection (cpp < 2 kHz) were not further analyzed and excluded from the average.

(1) $$G_{1\text{comp}}(\tau )=(1+\frac{T}{1-T}e^{\frac{-\tau }{\tau_{\text{tip}}}})\times \frac{1}{N\times (1+\frac{\tau }{\tau_{D}})\times \sqrt{1+\frac{\tau }{SP^{2}\tau_{D}}}}$$

For analysis of the FBS–AF647 series,^[52,53]^ data of free FBS–AF647 were first fitted with one component fits *G*_1comp_(*τ*). This yielded diffusion times for the randomly labeled proteins. All the data were then fitted with two-component fits *G*_2comp_(*τ*). The free protein diffusion time from above (FBS–AF647) was then fixed as *τ*_1_ corresponding to the free protein diffusion fraction. Diffusion times for nanoparticles obtained from measurements with 488 nm excitation and one-component fits were fixed (*τ*_2_) to represent the second component, which was the nanoparticle fraction. The two-component fits then yielded the % of each fraction (*F*_1_, *F*_2_; *N* = *n*1 + *n*2). *F*_2_ × 100 represented the % of particles, hence, the level of protein binding

(2) $$\begin{array}{lll} G_{2\text{comp}}(\tau )=(1+\frac{T}{1-T}e^{\frac{-\tau }{\tau_{\text{trip}}}})\times \frac{1}{N}\times \\ \; & \; & \left[\frac{F_{1}}{(1+\frac{\tau }{\tau_{1}})\times \sqrt{1+\frac{\tau }{SP^{2}\tau_{1}}}}+\frac{1-F_{1}}{(1+\frac{\tau }{\tau_{2}})\times \sqrt{1+\frac{\tau }{SP^{2}\tau_{2}}}}\right] \end{array}$$

### SPN Colloidal Stability Studies

See the Supporting Methods in the Supporting Information for all details of stability studies of all SPNs discussed in this work.

### IgG–BCN and Fab–BCN Synthesis

Both IgG–BCN and Fab–BCN were synthesized based on previously reported method.^[54]^ In a microtube (0.5 mL Eppendorf protein LoBind), Ontruzant IgG or Fab (100 μL, 20 μm in borate buffered saline (BBS) buffer (pH 8, 50 mm)), dibromopyridazinedione–PEG–BCN (6 μL, 10 mm in dimethyl sulfoxide (DMSO), see Figure S21 in the Supporting Information for structure), and tris(2-carboxyethyl)phosphine hydrochloride TCEP.HCL (3 μL, 10 mm in BBS buffer) were added. The resulting solution was left to react on an orbital shaker at room temperature for 6 h. Excess linker and TCEP were removed via centrifuge filtration (Amicon 10 kDa MWCO, 0.5 mL) for a total of six washes resuspending into 100 mm carbonate buffer at pH 9.8 each time. All solutions were stored at –20 °C until needed. IgG–BCN (75 μL, 17 μm, yield of 64%) and Fab–BCN (65 μL, 26 μm, yield of 85%) were obtained. IgG and Fab concentrations were measured using UV–vis (*A*_280 nm_ assuming ▯_(IgG)_ = 215 000 cm^–1^ and ▯_(Fab)_ = 70 000 cm^–1^).

### Affi–BCN

In a microtube (0.5 mL Eppendorf protein LoBind), affibody Z_HER2:2395_–Cys (130 μL, 20 μm in BBS buffer (pH 8, 50 mm)) and TCEP. HCL (1 μL, 10 mm in BBS buffer) were added. The solution was heated at 70 °C for 30 min. The solution was allowed to cool before adding monobromopyridazinedione–PEG–BCN (2.6 μL, 20 mm in DMSO) and was left to react on an orbital shaker at room temperature for 6 h. Excess of linker and TCEP were removed via centrifuge filtration (Amicon 3 kDa MWCO, 0.5 mL) for a total of six washes resuspending into 100 mm carbonate buffer at pH 9.8 each time. Affi–BCN (75 μL, 34 μm, yield of 97%) was obtained. Affibody concentrations were measured using UV–vis (using the predicted ▯ = 8400 cm^–1^ at *A*_280 nm_). Electrospray ionisation mass spectrometry (MS (ESI)): measured 7495.4 Da, calculated 7497.8 Da (see the Supporting Methods in the Supporting Information for further details). Solution was stored at –20 °C until needed.

### SPN–IgG Synthesis

In a microtube (0.5 mL Eppendorf protein LoBind), SPN–PEG_2k_–N_3_ (70 μL, 113 nm in carbonate buffer (pH 9.8, 100 mm)) and IgG–BCN (26 μm, 152 μL in carbonate buffer) were added. The resulting solution was left to react on an orbital shaker at room temperature for 18 h. Tween-20 (30 μL, 0.5 v/v% in water) was added to prevent nanoparticles from sticking to the filters. Sample was concentrated to <100 μL by centrifuge filtration (Amicon 100 kDa MWCO, 9000 rpm for 2 min). The concentrated sample was run through a column (Sephacryl 300-HR) and multiple 0.5 mL aliquots were recovered. Fluorescent fractions were then combined and further Tween-20 was added (so that the final Tween-20 content was 0.05 v/v%). Combined fractions were then concentrated by centrifuge filtration. The resulting yellow solution was stored at 4 °C until needed (160 μL, 6.1 nm, yield of 12%).

### SPN–Fab Synthesis

In a microtube (0.5 mL Eppendorf Protein LoBind), SPN–PEG_2_k–N_3_ (18 μL, 113 nm in carbonate buffer (pH 9.8, 100 mm)) and Fab–BCN (25 μm, 82 μL in carbonate buffer) were added. The solution was left to react on an orbital shaker at room temperature for 18 h. Solution was diluted to 500 μL with PBS with Tween-20 0.05 v/v% (PBST) and excess Fab–BCN in solution was removed via centrifuge filtration (Amicon 100 kDa MWCO, 0.5 mL) for a total of five washes resuspending into PBST each time. The resulting yellow solution was stored at 4 °C until needed (60 μL, 14 nm, yield of 41%).

### SPN–Affi Synthesis

In a microtube (0.5 mL Eppendorf Protein LoBind), SPN–PEG2k–N3 (27 μL, 113 nm in carbonate buffer (pH 9.8, 100 mm)) and Affi–BCN (20 μm, 153 μL in carbonate buffer) were added. The solution was left to react on an orbital shaker at room temperature for 18 h. Solution was diluted to 500 μL with PBST and excess Affi–BCN in solution was removed via centrifuge filtration (Amicon 100 kDa MWCO, 0.5 mL) for a total of five washes resuspending into PBST each time. The resulting yellow solution was stored at 4 °C until needed (60 μL, 23.3 nm, yield of 46%).

### LFIA and FLISA

See the Supporting Methods in the Supporting Information for all details of LFIA and FLISA work.

### Cell Culture

Human ovarian adenocarcinoma cell line SKOV3 (HER2+), breast adenocarcinoma cell line MDA-MB-468 (HER2–), and epithelial cell line HEK-293 were grown as monolayers in Dulbecco’s modified Eagle medium (high glucose) with Glutamax (Gibco, Carlsbad, CA, USA) supplemented with 10 v/v% fetal calf serum (Gibco, Carlsbad, CA, USA) and maintained at 5% CO_2_. Cell viability was assessed by trypan blue exclusion.

### Flow Cytometry

Live cells (SKOV3 and MDA-MB-468) were resuspended in a single cell suspension following trypsinization (0.05 v/v% trypsin ethylenediaminetetraacetic acid (EDTA), Gibco). Cells were incubated with SPN–PEG_2000_–N_3_ (SPN-Control), IgG–SPN, Fab–SPN, and Affi–SPN (at a concentration of 1 nm in PBS) for 1 h at 4 °C in flow cytometry staining buffer (PBS, EDTA 1 mm, N-2-hydroxyethylpiperazine-N′-2-ethanesulfonic acid (HEPES) 25 mm, FBS 1 v/v%). For the “blocked” control, SKOV3 cells were preincubated with Ontruzant (for IgG and Fab–SPN) and Affibody Z_HER2:2395_–Cys (for Affi–SPN incubation) for 10 min at 4 °C in flow cytometry staining buffer (at a concentration of 20 nm) followed by incubation with IgG–SPN, Fab–SPN, and Affi–SPN, as above. Each suspension was analyzed using a BD LRSFortessa and the data were processed using FlowJo software (Ashland).

### Cytotoxicity (MTT Assay)

SPN cytotoxicity was assessed by using a 3-(4,5-dimethylthiazol-2-yl)-2-5-diphenyltetrazolium bromide (MTT) assay. HEK-293 cells were seeded in 384 well plates and treated the following day with SPNs (SPN–PEG_2000_–N_3_ (SPN-Control), IgG–SPN, Fab–SPN, and Affi–SPN) at different concentrations (20, 10, 5, 2, and 1 nm). The MTT assay was performed 72 h postincubation, in which MTT (molecular probes, life technologies, 5 mg mL^–1^) was incubated with the cells at 37 °C for 2 h. Blue formazan salts were dissolved in DMSO and the absorbance (570 nm) was read spectrophotometrically.

### Immunofluorescence Staining and Confocal Imaging

SKOV3 cells were seeded at a concentration of 1 50 000 cells mL^–1^ in 8 well ibidi plates (ibidi GmbH, Germany). The following day, cells were fixed with paraformaldehyde 4 v/v% for 10 min and washed with PBS. Cells were then incubated with SPN–PEG_2000_–N_3_ (SPN-Control) and SPN–Affi (1 nm) overnight at 4 °C in PBS. The plates were then washed with further PBS and then counterstained with 4′,6-diamidino-2-phenylindole (DAPI). Images were taken using a Leica SP8 inverted confocal microscope, using Leica Las X software and then reconstructed with ImageJ/Fiji (NIH, USA).

### Photostability

See the Supporting Methods in the Supporting Information for all details of photostability experiments.

### Zebrafish Circulation and Xenograft Targeting

See the Supporting Methods in the Supporting Information for all details of zebrafish experiments.

### Statistical Analysis

All statistical analyses were performed in Prism. The specifics associated with preprocessing of data, sample sizes, and statistical methods, including post-hoc test methods, were given in the respective figure captions. All box plots displayed the 5th and 95th percentile values (top and bottom horizontal lines), the lower quartile (lower boundary of the box), the median (the line inside the box), and the upper quartile (upper boundary of the box). All relevant values were displayed as mean ± standard deviation (S.D.) (unless stated otherwise).

## Supplementary Material

Supporting information

## Data Availability

The data that support the findings of this study are available from the corresponding author upon reasonable request.

## References

[1] Li J, Rao J, Pu K (2018). Biomaterials.

[2] Li J, Pu K (2020). Acc Chem Res.

[3] Yang Z, Li L, Jin AJ, Huang W, Chen X (2020). Mater Horiz.

[4] Cui D, Huang J, Zhen X, Li J, Jiang Y, Pu K (2019). Angew Chem Int Ed.

[5] Li J, Huang J, Lyu Y, Huang J, Jiang Y, Xie C, Pu K (2019). J Am Chem Soc.

[6] Yang Z, Fan W, Tang W, Shen Z, Dai Y, Song J, Wang Z, Liu Y, Lin L, Shan L, Liu Y (2018). Angew Chem.

[7] Hu X, Tang Y, Hu Y, Lu F, Lu X, Wang Y, Li J, Li Y, Ji Y, Wang W, Ye D (2019). Theranostics.

[8] Xie C, Zhou W, Zeng Z, Fan Q, Pu K (2020). Chem Sci.

[9] Xie C, Zhen X, Lei Q, Ni R, Pu K (2017). Adv Funct Mater.

[10] Xie C, Zhen X, Miao Q, Lyu Y, Pu K (2018). Adv Mater.

[11] Islam R, Maeda H, Fang J (2022). Expert Opin Drug Delivery.

[12] Mittelheisser V, Coliat P, Moeglin E, Goepp L, Goetz JG, Charbonnière LJ, Pivot X, Detappe A (2022). Adv Mater.

[13] Wang D, Liu J, Liu Z, Zhang Z, Sun Z, Wu C, Wang G (2020). ACS Appl Nano Mater.

[14] Wu C, Schneider T, Zeigler M, Yu J, Schiro PG, Burnham DR, McNeill JD, Chiu DT (2010). J Am Chem Soc.

[15] Rong Y, Wu C, Yu J, Zhang X, Ye F, Zeigler M, Gallina ME, Wu IC, Zhang Y, Chan YH, Sun W (2013). ACS Nano.

[16] Sgro C (1995). Toxicology.

[17] Chen S, Florinas S, Teitgen A, Xu ZQ, Gao C, Wu H, Kataoka K, Cabral H, Christie RJ (2017). Sci Technol Adv Mater.

[18] Richards DA, Maruani A, Chudasama V (2016). Chem Sci.

[19] Marques AC, Costa PJ, Velho S, Amaral MH (2020). J Controlled Release.

[20] Sapra P, Moase EH, Ma J, Allen TM (2004). Clin Cancer Res.

[21] Duan D, Wang A, Ni L, Zhang L, Yan X, Jiang Y, Mu H, Wu Z, Sun K, Li Y (2018). Int J Nanomed.

[22] Ruigrok VJB, Levisson M, Eppink MHM, Smidt H, van der Oost J (2011). Biochem J.

[23] Grönwall C, Stahl S (2009). J Biotechnol.

[24] Richards DA (2018). Drug Discovery Today: Technol.

[25] Kramer-Marek G, Kiesewetter DO, Martiniova L, Jagoda E, Lee SB, Capala J (2008). Eur J Nucl Med Mol Imaging.

[26] Mączyńska J, Da Pieve C, Burley TA, Raes F, Shah A, Saczko J, Harrington KJ, Kramer-Marek G (2020). Cell Death Dis.

[27] Gao J, Chen K, Miao Z, Ren G, Chen X, Gambhir SS, Cheng Z (2011). Biomaterials.

[28] Jokerst JV, Miao Z, Zavaleta C, Cheng Z, Gambhir SS (2011). Small.

[29] Narsireddy A, Vijayashree K, Adimoolam MG, Manorama SV, Rao NM (2015). Int J Nanomed.

[30] Cheng K, Chen H, Jenkins CH, Zhang G, Zhao W, Zhang Z, Han F, Fung J, Yang M, Jiang Y, Xing L (2017). ACS Nano.

[31] Feng G, Fang Y, Liu J, Geng J, Ding D, Liu B (2017). Small.

[32] Liu J, Feng G, Ding D, Liu B (2013). Polym Chem.

[33] Dal NJK, Kocere A, Wohlmann J, Van Herck S, Bauer TA, Resseguier J, Bagherifam S, Hyldmo H, Barz M, De Geest BG, Fenaroli F (2020). Small.

[34] Kocere A, Resseguier J, Wohlmann J, Skjeldal FM, Khan S, Speth M, Dal NJK, Ng MYW, Alonso-Rodriguez N, Scarpa E, Rizzello L (2020). EBioMedicine.

[35] Bourke S, Donà F, Teijeiro Gonzalez Y, Qazi Chaudhry B, Panamarova M, Mackay E, Zammit PS, Dailey LA, Eggert US, Suhling K, Green MA (2022). ACS Appl Polym Mater.

[36] Sun J, Chen N, Chen X, Zhang Q, Gao F (2019). Anal Chem.

[37] Qian CG, Zhu S, Feng PJ, Chen YL, Yu JC, Tang X, Liu Y, Shen QD (2015). ACS Appl Mater Interfaces.

[38] Yang D, Zhang S, Hu Y, Chen J, Bao B, Yuwen L, Weng L, Cheng Y, Wang L (2016). RSC Adv.

[39] Lubanska D, Alrashed S, Mason GT, Nadeem F, Awada A, DiPasquale M, Sorge A, Malik A, Kojic M, Soliman MAR, deCarvalho AC (2022). Sci Rep.

[40] Cong S, Creamer A, Fei Z, Hillman SAJ, Rapley C, Nelson J, Heeney M (2020). Macromol Biosci.

[41] Creamer A, Wood CS, Howes PD, Casey A, Cong S, Marsh AV, Godin R, Panidi J, Anthopoulos TD, Burgess CH, Wu T (2018). Nat Commun.

[42] Wang C, Liu F, Chen QM, Xiao CY, Wu YG, Li WW (2021). Chin J Polym Sci.

[43] Rimmele M, Glöcklhofer F, Heeney M (2022). Mater Horiz.

[44] Chan Y-H, Wu P-J (2015). Part Part Syst Charact.

[45] Zhang D, Wu M, Zeng Y, Liao N, Cai Z, Liu G, Liu X, Liu J (2016). J Mater Chem B.

[46] Pu K, Shuhendler AJ, Valta MP, Cui L, Saar M, Peehl DM, Rao J (2014). Adv Healthcare Mater.

[47] Pu K, Mei J, Jokerst JV, Hong G, Antaris AL, Chattopadhyay N, Shuhendler AJ, Kurosawa T, Zhou Y, Gambhir SS, Bao Z (2015). Adv Mater.

[48] Zhen X, Pu K, Jiang X (2021). Small.

[49] Shou K, Tang Y, Chen H, Chen S, Zhang L, Zhang A, Fan Q, Yu A, Cheng Z (2018). Chem Sci.

[50] Pu K, Shuhendler AJ, Rao J (2013). Angew Chem.

[51] Alberg I, Kramer S, Schinnerer M, Hu Q, Seidl C, Leps C, Drude N, Möckel D, Rijcken C, Lammers T, Diken M (2020). Small.

[52] Najer A, Blight J, Ducker CB, Gasbarri M, Brown JC, Che J, Høgset H, Saunders C, Ojansivu M, Lu Z, Lin Y (2022). ACS Cent Sci.

[53] Najer A, Belessiotis-Richards A, Kim H, Saunders C, Fenaroli F, Adrianus C, Che J, Tonkin RL, Høgset H, Lörcher S, Penna M (2022). Small.

[54] Nogueira JCF, Paliashvili K, Bradford A, Di Maggio F, Richards DA, Day RM, Chudasama V (2020). Org Biomol Chem.

[55] Bahou C, Chudasama V (2022). Org Biomol Chem.

[56] Campbell F, Bos FL, Sieber S, Arias-Alpizar G, Koch BE, Huwyler J, Kros A, Bussmann J (2018). ACS Nano.

[57] Chen YY, Syed AM, MacMillan P, Rocheleau JV, Chan WCW (2020). Adv Mater.

[58] Yang Z, Zheng S, Harrison WJ, Harder J, Wen X, Gelovani JG, Qiao A, Li C (2007). Biomacromolecules.

[59] Lobatto ME, Calcagno C, Millon A, Senders ML, Fay F, Robson PM, Ramachandran S, Binderup T, Paridaans MPM, Sensarn S, Rogalla S (2015). ACS Nano.

[60] Müller P, Schwille P, Weidemann T (2014). Bioinformatics.

[61] Kapusta P (2010). Absolute Diffusion Coefficients: Compilation of Reference Data for FCS Calibration.

