# Supplementary material for: Modular Synthesis of Semiconducting Graft Copolymers to Achieve “Clickable” Fluorescent Nanoparticles with Long Circulation and Specific Cancer Targeting
Source: Adv Mater. Author manuscript; Available in PMC 2024 Dec 8. (PMC7616993; doi:10.1002/adma.202300413)
Supplement: Supporting information [file EMS193199-supplement-Supporting_information.pdf]

ADVANCED MATERIALS

Supporting Information

for *Adv. Mater.,* DOI: 10.1002/adma.202300413

Modular Synthesis of Semiconducting Graft Copolymers to Achieve "Clickable" Fluorescent Nanoparticles with Long Circulation and Specific Cancer Targeting

*Adam Creamer, Alessandra Lo Fiego, Alice Agliano, Lino Prados-Martin, Håkon Høgset, Adrian Najer, Daniel A. Richards, Jonathan P. Wojciechowski, James E. J. Foote, Nayoung Kim, Amy Monahan, Jiaqing Tang, André Shamsabadi, Léa N. C. Rochet, Ioanna A. Thanasi, Laura R. de la Ballina, Charlotte L. Rapley, Stephen Turnock, Elizabeth A. Love, Laurence Bugeon, Margaret J. Dallman, Martin Heeney, Gabriela Kramer-Marek, Vijay Chudasama, Federico Fenaroli, and Molly M. Stevens**

Modular synthesis of semiconducting graft co-polymers to achieve 'clickable' fluorescent nanoparticles with long circulation and specific cancer targeting

*Adam Creamer,[a] Alessandra Lo Fiego,[a] Alice Agliano,[a] Lino Prados-Martin,[a] Håkon Høgset,[a] Adrian Najer,[a] Daniel A. Richards,[a] Jonathan P. Wojciechowski,[a] James E.J. Foote,[a] Nayoung Kim,[a] Amy Monahan,[a] Jiaqing Tang,[a] André Shamsabadi,[a] Léa N. C. Rochet,[b] Ioanna A. Thanasi,[b] Laura R. de la Ballina,[c,d] Charlotte L. Rapley,[e] Stephen Turnock,[f] Elizabeth A. Love,[g] Laurence Bugeon,[h] Margaret J. Dallman,[h] Martin Heeney,[e] Gabriela Kramer-Marek,[f] Vijay Chudasama,[b] Federico Fenaroli,[i,j] Molly M. Stevens [a]**

Supplementary Figures **2**
Supplementary Methods **32**

Supplementary Figures

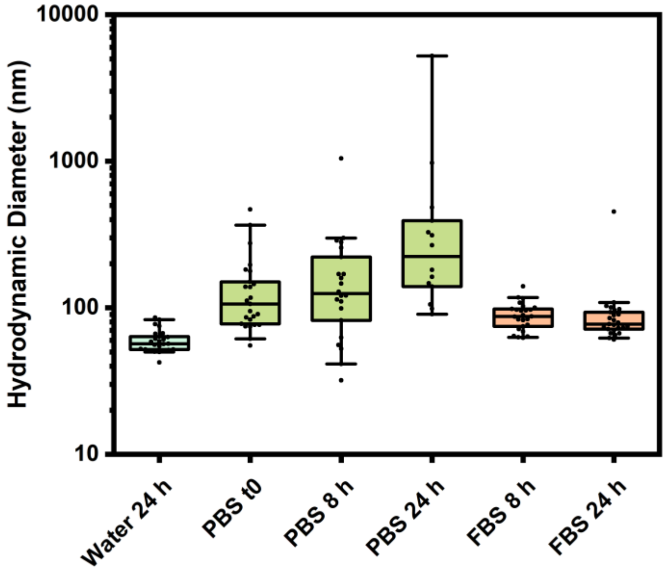

Figure S1. Hydrodynamic diameter of SPN-PEG_{750} in water, PBS (at room temperature) and 10 v/v% FBS (in PBS, at 37 °C) at various timepoints calculated from FCS autocorrelation curves (N = 1, n ≥ 14 technical replicates). Box plots: 5th and 95th percentile value (top and bottom horizontal lines), the lower quartile (lower boundary of the box), the median (the line inside the box) and the upper quartile (upper boundary of the box).

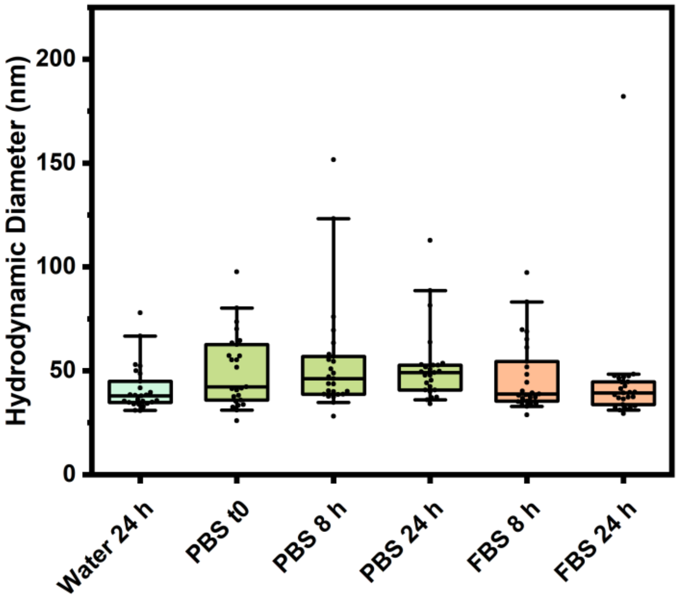

Figure S2. Hydrodynamic diameter of SPN-PEG$_{2000}$ in water, PBS (at room temperature) and 10 v/v% FBS (in PBS, at 37 °C) at various timepoints calculated from FCS autocorrelation curves (n = 25 technical replicates). Box plots: 5th and 95th percentile value (top and bottom horizontal lines), the lower quartile (lower boundary of the box), the median (the line inside the box) and the upper quartile (upper boundary of the box).

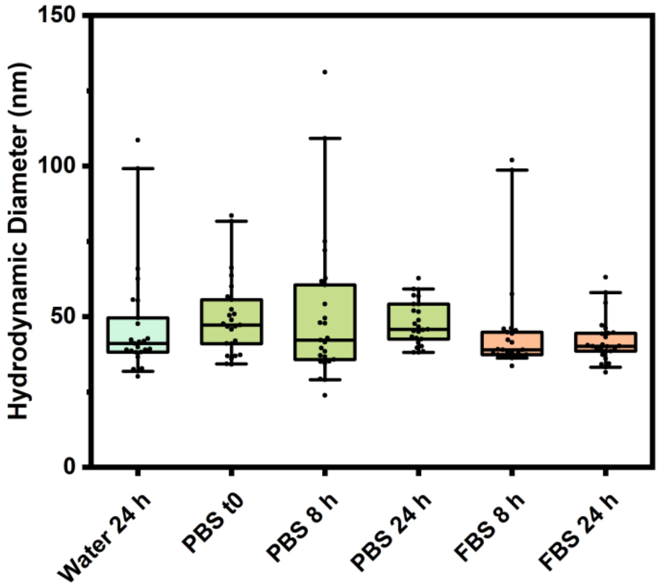

Figure S3. Hydrodynamic diameter of SPN-PEG_{5000} in water, PBS (at room temperature) and 10 v/v% FBS (in PBS, at 37 °C) at various timepoints calculated from FCS autocorrelation curves (n = 25 technical replicates). Box plots: 5th and 95th percentile value (top and bottom horizontal lines), the lower quartile (lower boundary of the box), the median (the line inside the box) and the upper quartile (upper boundary of the box).

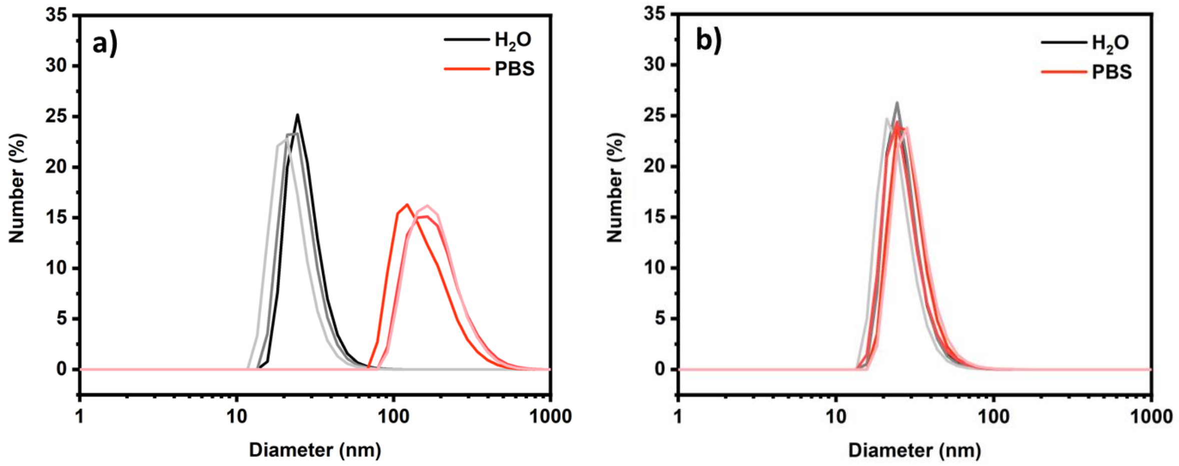

Figure S4. DLS number distributions for a) SPN-PEG_{750} and b) SPN-PEG_{5000} in water and PBS after 24 hours. SPN-PEG_{2000} number distributions are shown in Figure 1c (n = 3 technical replicates).

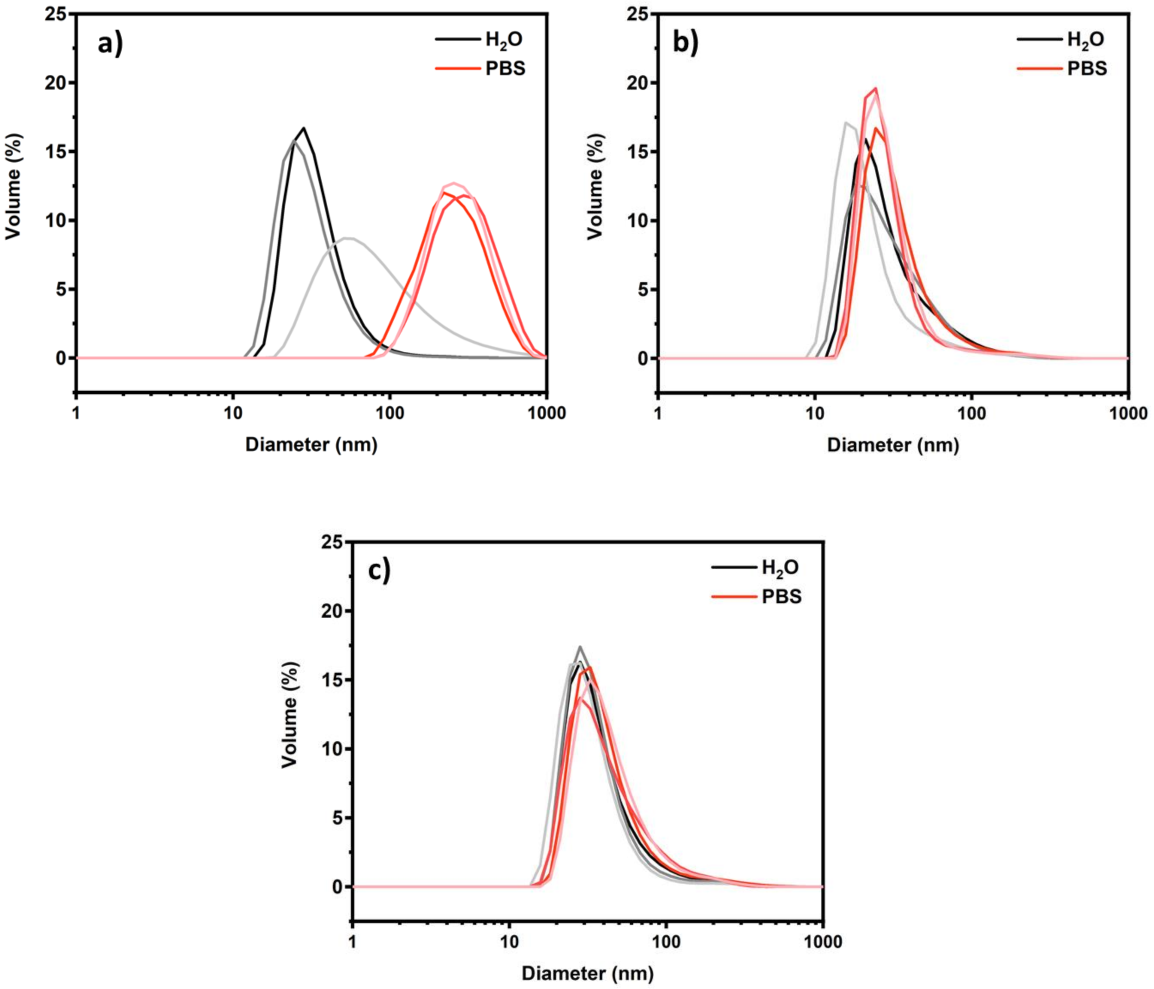

Figure S5. DLS volume distributions for a) SPN-PEG$_{750}$, b) SPN-PEG$_{2000}$ and c) SPN-PEG$_{5000}$ in water and PBS after 24 hours (n = 3 technical replicates).

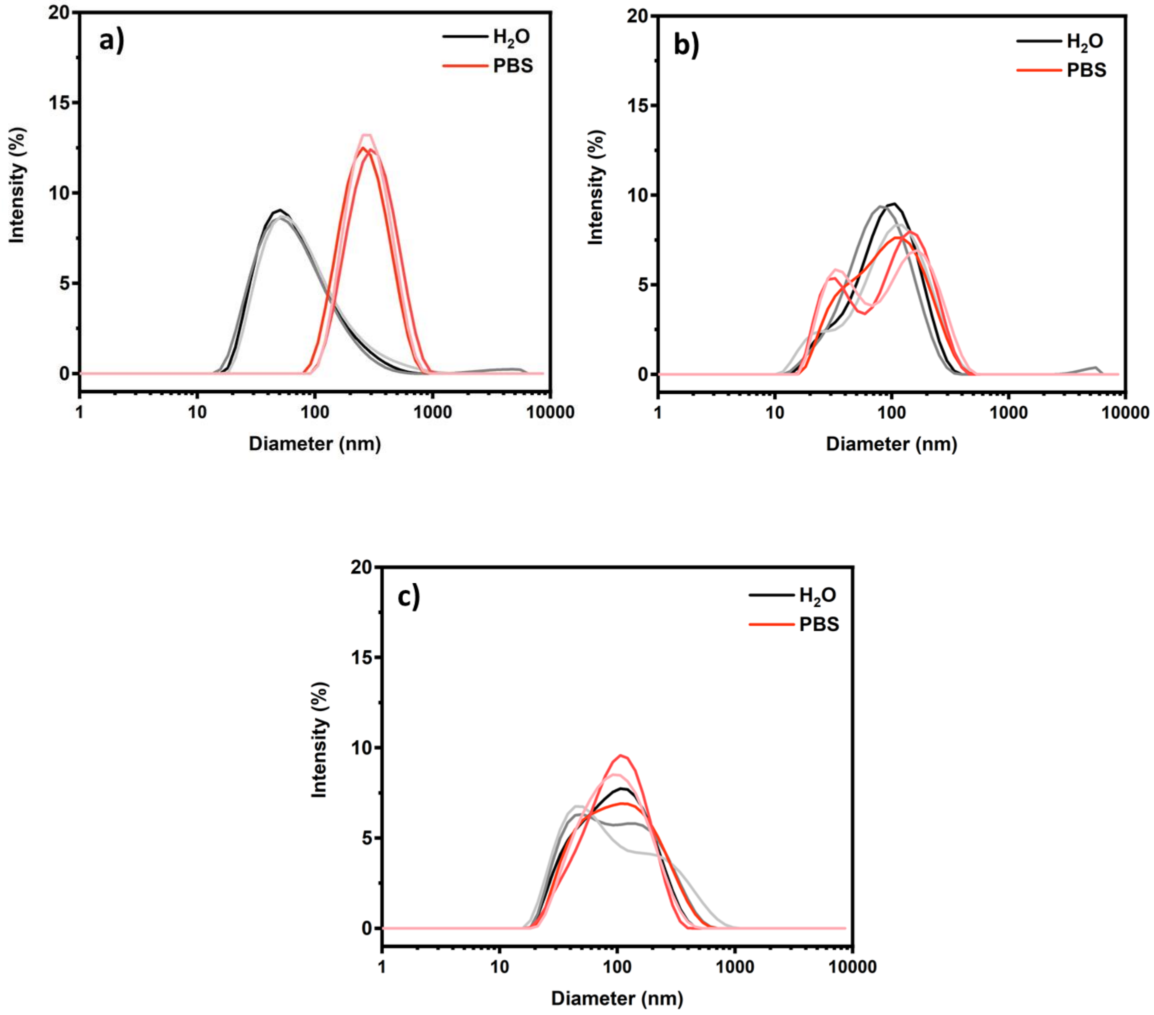

Figure S6. DLS intensity distributions for a) SPN-PEG$_{750}$, b) SPN-PEG$_{2000}$ and c) SPN-PEG$_{5000}$ in water and PBS after 24 hours (n = 3 technical replicates).

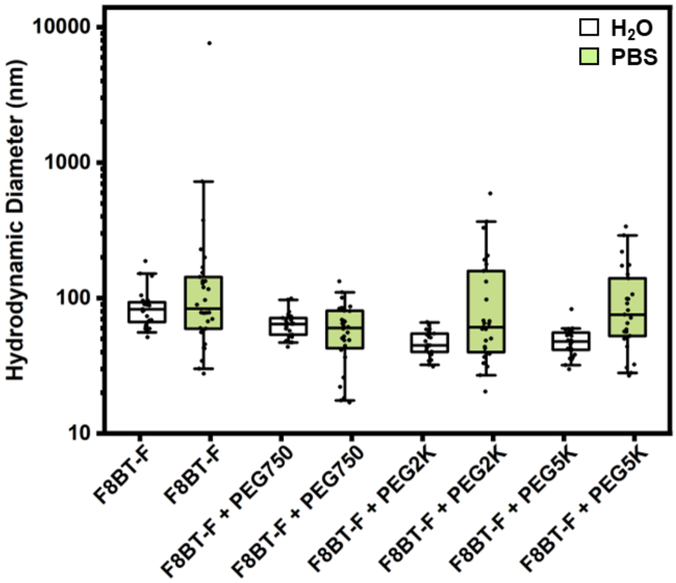

Figure S7. Hydrodynamic diameter of SPNs generated by co-precipitation of F8BT and PEG in water and PBS after 8 hours, calculated from FCS autocorrelation curves ($n \geq 25$ technical replicates). Box plots: 5th and 95th percentile value (top and bottom horizontal lines), the lower quartile (lower boundary of the box), the median (the line inside the box) and the upper quartile (upper boundary of the box).

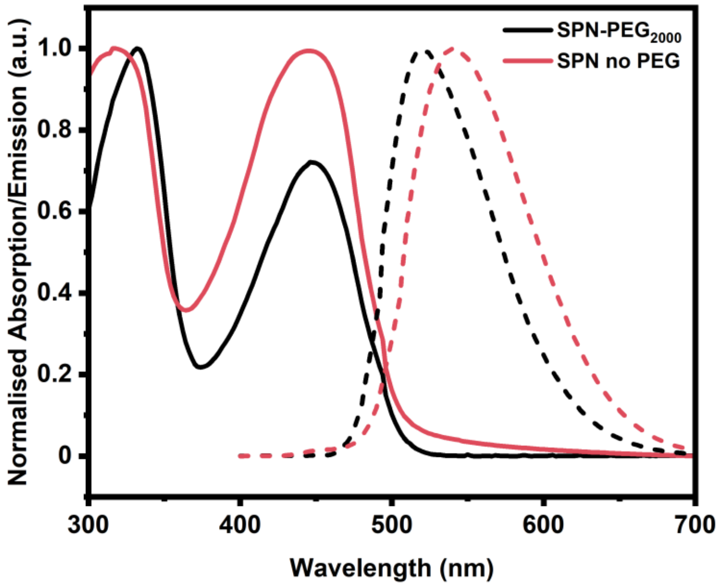

Figure S8. *Normalised absorption (solid line) and fluorescence spectra (dotted line, λ_{Ex} = 450 nm) of SPN-PEG$_{2000}$ and bare F8BT-F SPNs (SPN no PEG) in water.*

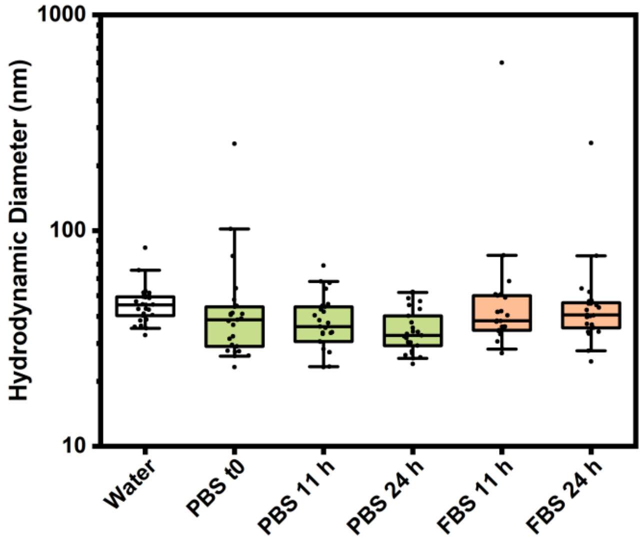

Figure S9. Hydrodynamic diameter of SPN-DSPE$_{2000}$ in water, PBS (at room temperature) and 10 v/v% FBS (in PBS, at 37 °C) at various timepoints calculated from FCS autocorrelation curves (n ≥ 25 technical replicates). Box plots: 5th and 95th percentile value (top and bottom horizontal lines), the lower quartile (lower boundary of the box), the median (the line inside the box) and the upper quartile (upper boundary of the box).

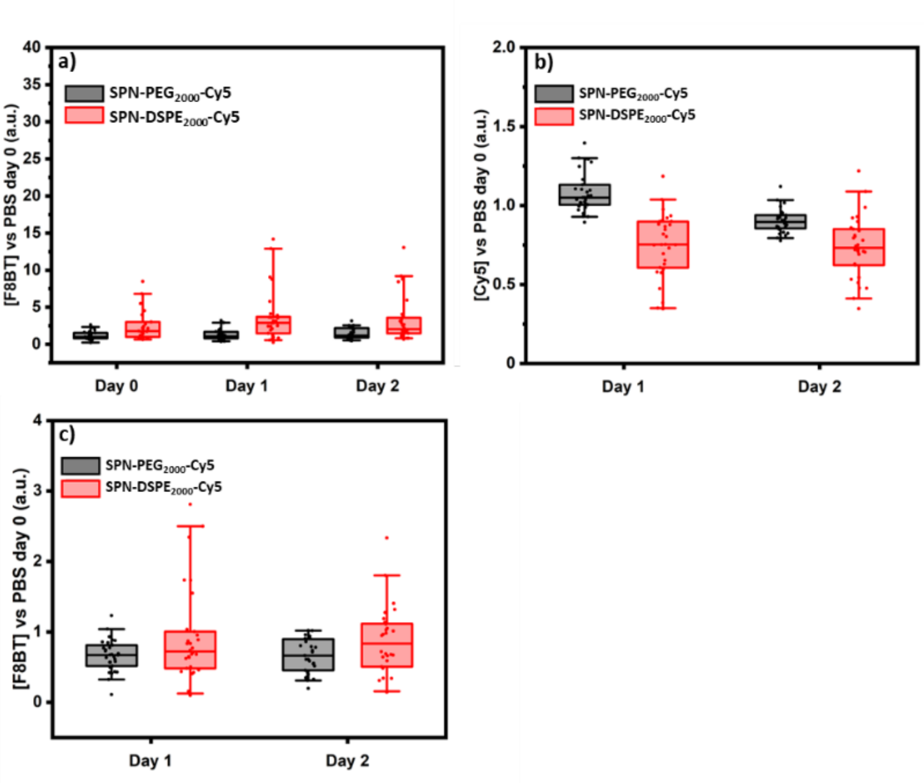

Figure S10. *FCS analysis of SPNs (with cy5 labelled PEG chains). Reported values are the concentration changes in the a) green channel (F8BT fluorescence) in FBS, b) red channel (cy5 fluorescence) in PBS and c) green channel in PBS. All measurements are made with respect to an initial measurement in PBS. All FCS data above is N = 1, n = 30 technical replicates. Box plots: 5th and 95th percentile value (top and bottom horizontal lines), the lower quartile (lower boundary of the box), the median (the line inside the box) and the upper \quartile (upper boundary of the box).*

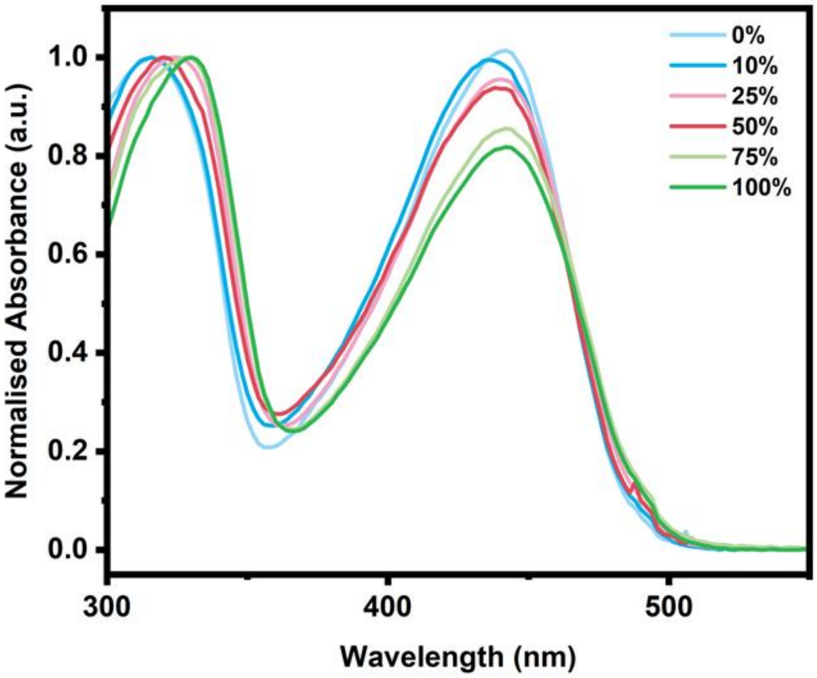

Figure S11. Normalised UV-vis absorption spectra of F8BT-PEG$_{2000}$ with a varied PEG grafting density in chloroform.

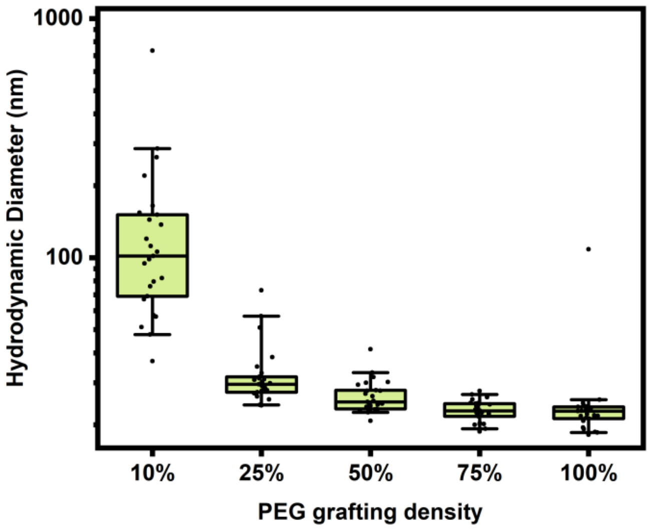

Figure S12. Hydrodynamic diameter of SPN-PEG_{2000} with a varied grafting density in when initially diluted into PBS, calculated from FCS autocorrelation curves (n = 25 technical replicates). Box plots: 5th and 95th percentile value (top and bottom horizontal lines), the lower quartile (lower boundary of the box), the median (the line inside the box) and the upper quartile (upper boundary of the box).

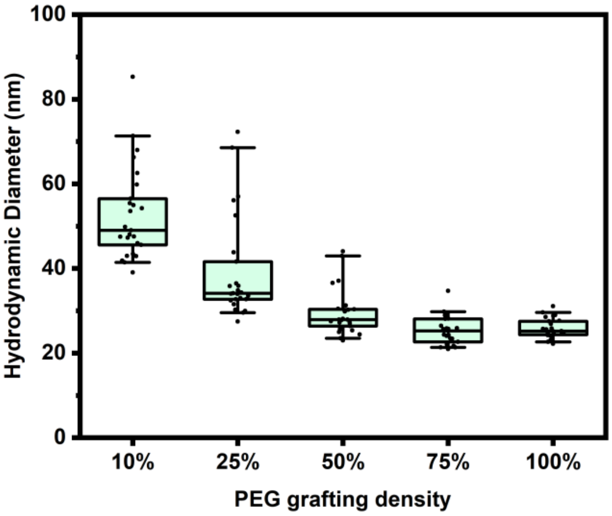

Figure S13. Hydrodynamic diameter of SPN-PEG_{2000} with a varied grafting density in water after 24 hours, calculated from FCS autocorrelation curves (n = 25 technical replicates). Box plots: 5th and 95th percentile value (top and bottom horizontal lines), the lower quartile (lower boundary of the box), the median (the line inside the box) and the upper quartile (upper boundary of the box).

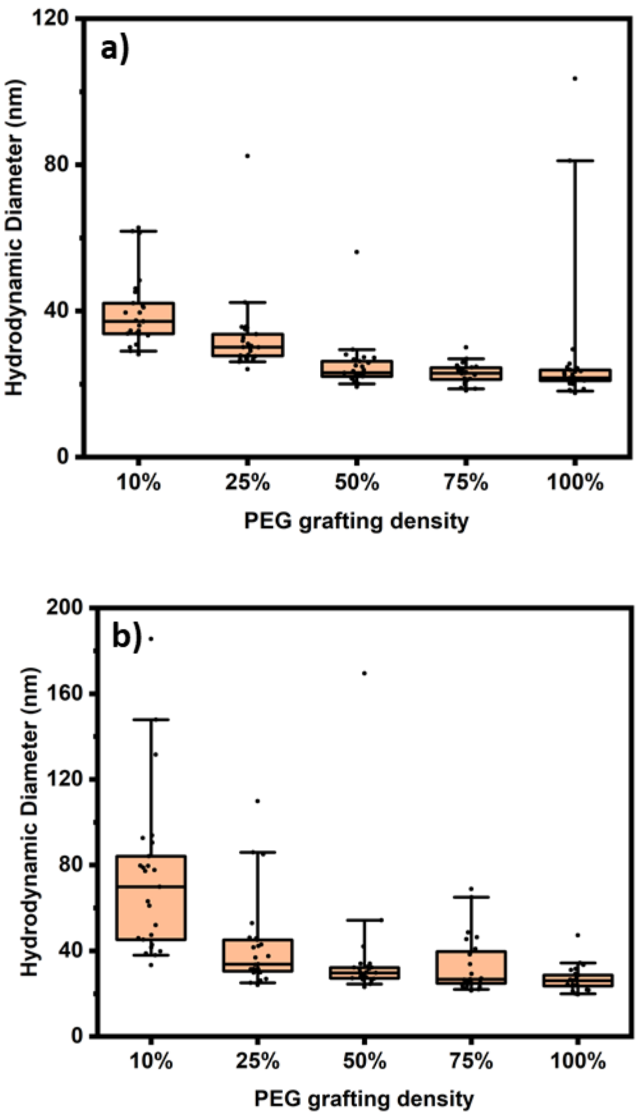

Figure S14. Hydrodynamic diameter of SPN-PEG_{2000} with a varied grafting density in 10 v/v% FBS (in PBS, at 37 °C), a) initial measurements and b) after 24 hours, calculated from FCS autocorrelation curves (n = 25 technical replicates). Box plots: 5th and 95th percentile value (top and bottom horizontal lines), the lower quartile (lower boundary of the box), the median (the line inside the box) and the upper quartile (upper boundary of the box).

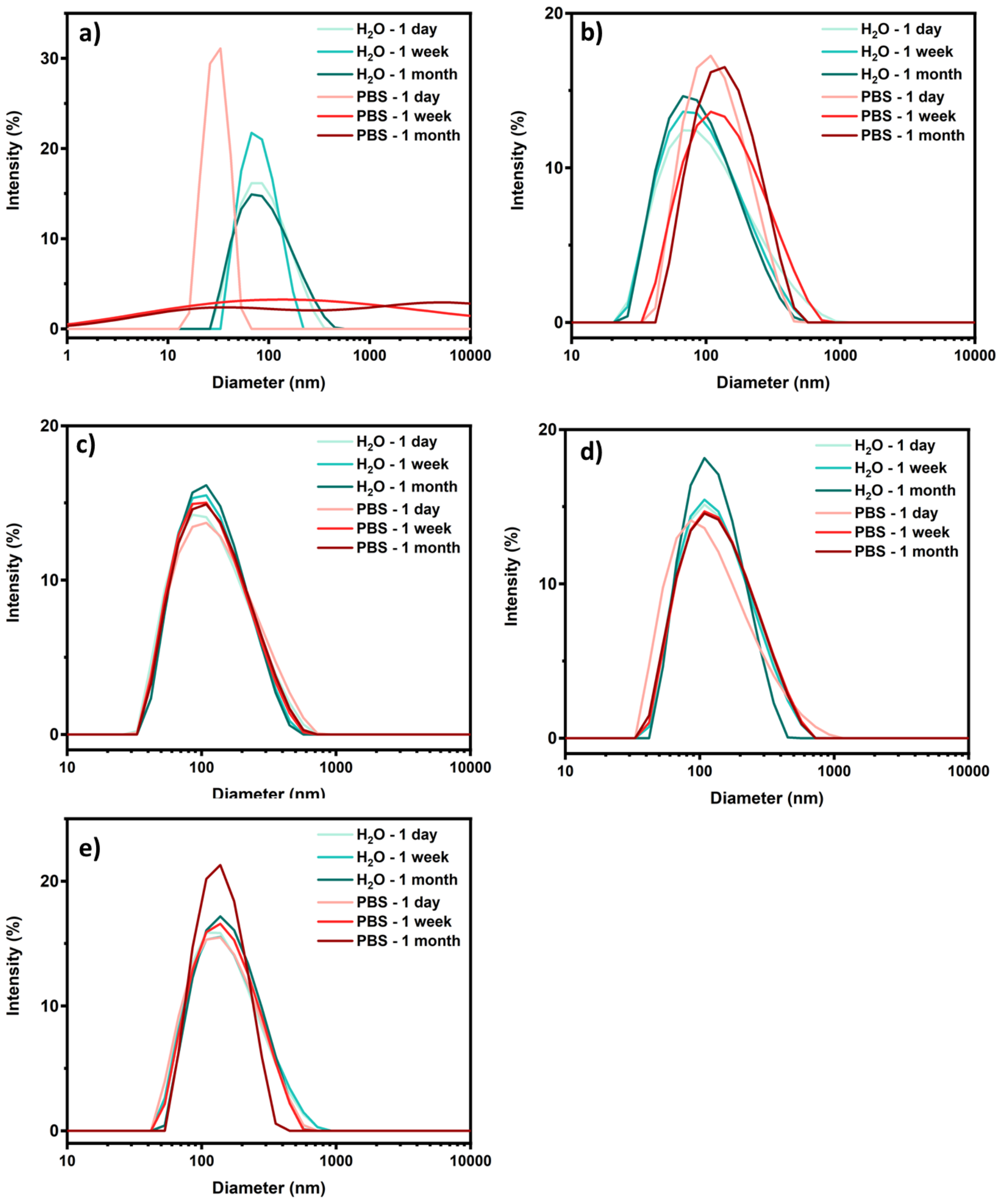

Figure S15. DLS intensity distributions of SPN-PEG$_{2000}$ with a varied PEG grafting density, in PBS and water at various timepoints. a) 10%, b) 25%, c) 50%, d) 75% and e) 100% (n = 1).

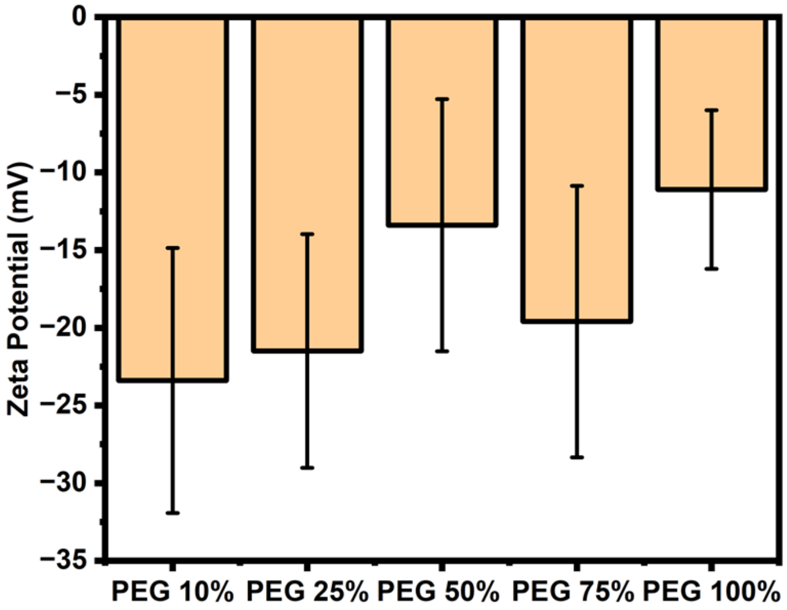

Figure S16. Zeta potential of SPN-PEG_{2000} with a varied PEG grafting density. Data are presented as mean ± standard deviation (n = 3).

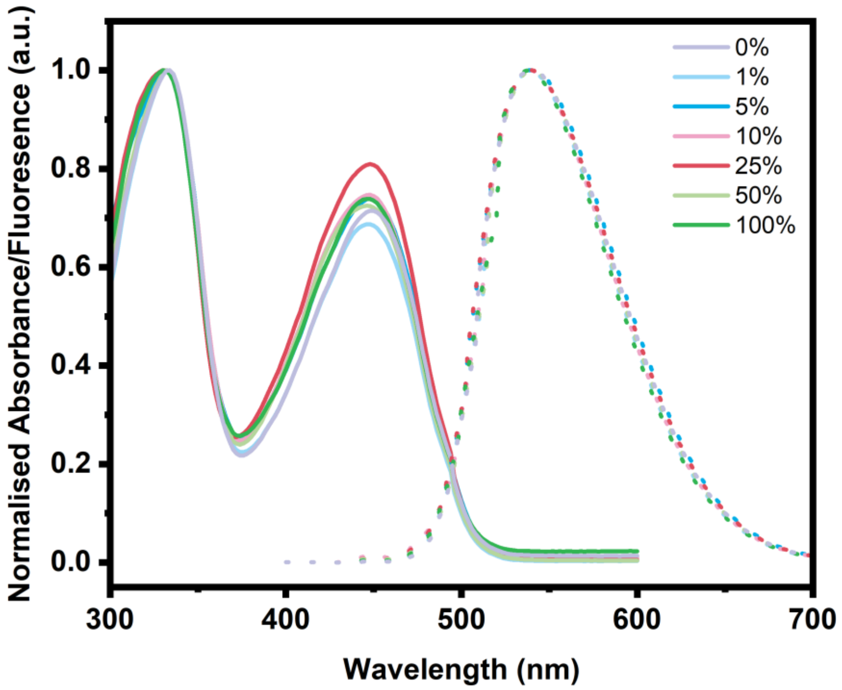

Figure S17. a) Normalised UV-Vis (solid line) and PL (dotted line) spectra of SPN-PEG$_{2000}$-N$_3$ with varied percentage azide content.

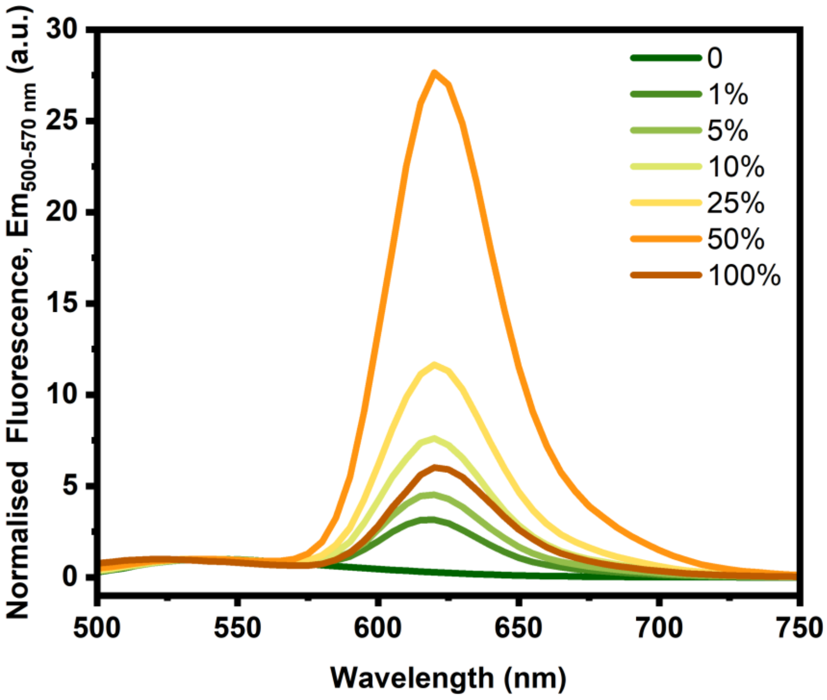

Figure S18. PL spectra (normalised to the donor emission maxima (500-570 nm)) of SPN-PEG$_{2000}$-N$_3$ with varied percentage azide content, functionalised with an excess of DBCO-AF594.

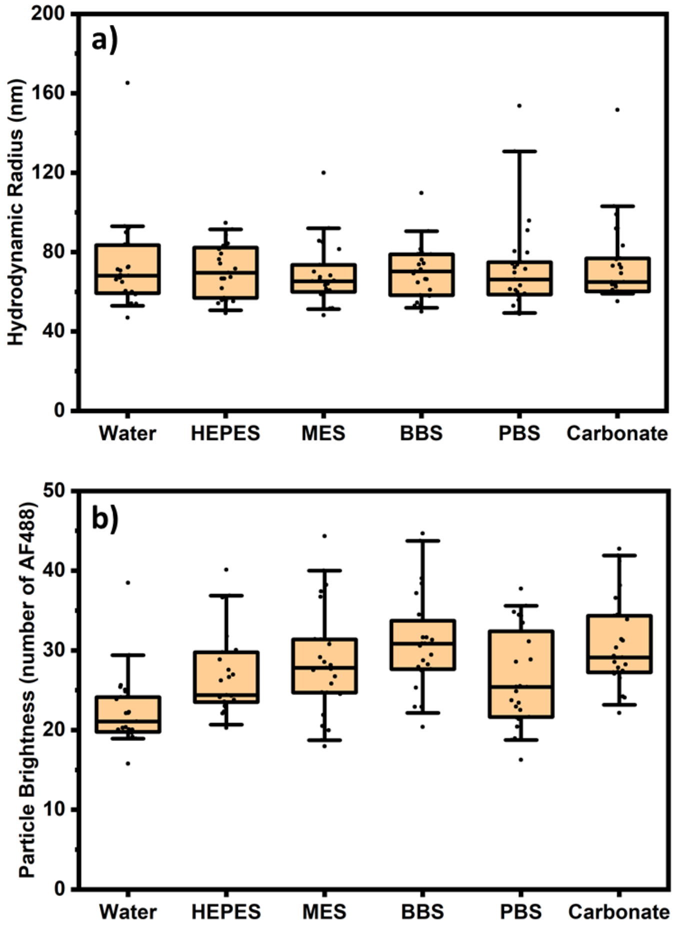

Figure S19. a) Hydrodynamic diameter and b) particle brightness (compared to AF488) in various buffer solutions after four days at room temperature. Values calculated from FCS analysis (n = 25 technical replicates). Box plots: 5th and 95th percentile value (top and bottom horizontal lines), the lower quartile (lower boundary of the box), the median (the line inside the box) and the upper quartile (upper boundary of the box).

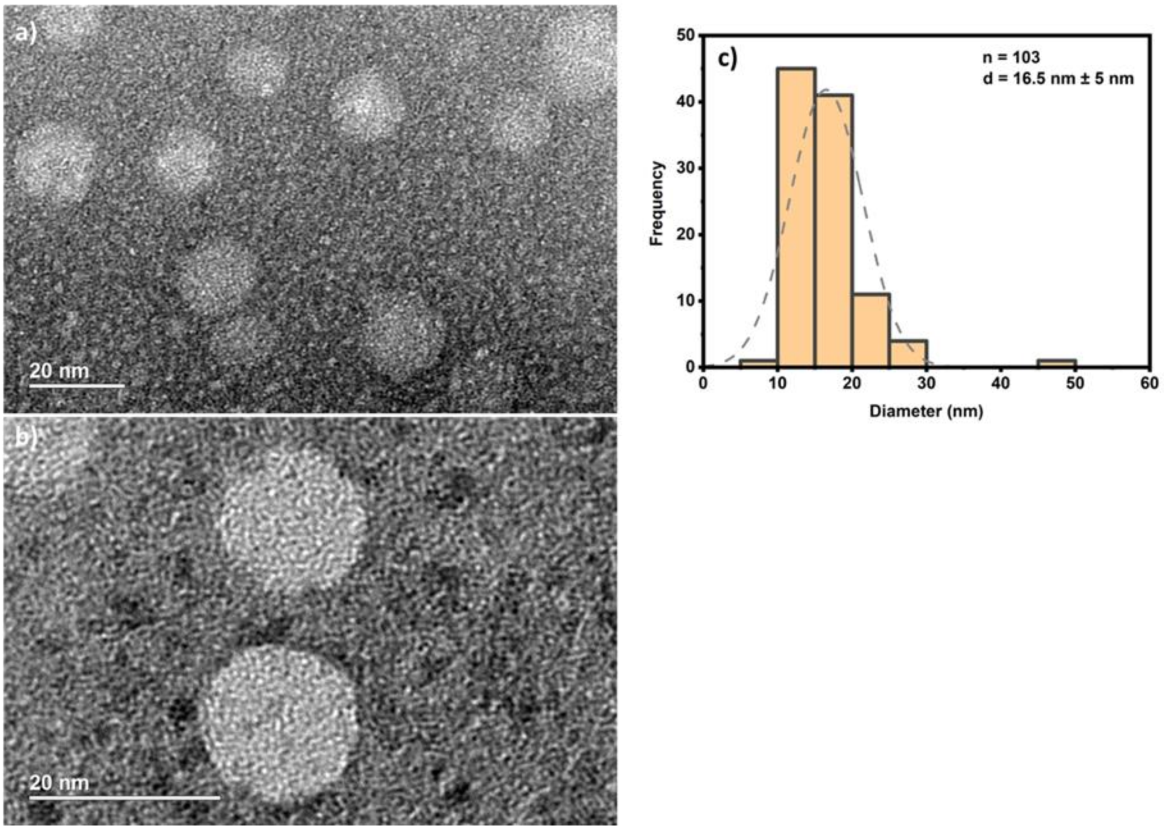

Figure S20. a, b) Representative TEM images of SPN-PEG$_{2000}$-N$_3$ with uranyl acetate negative staining. c) Histogram of diameter measurements from TEM analysis (n = 103 particle count).

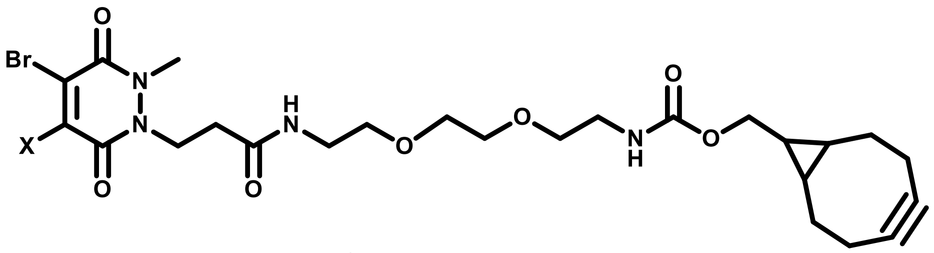

X = H :- Monobromo-PD-BCN
X = Br :- Dibromo-PD-BCN

Figure S21. Chemical structure of mono and dibromo-PD linkers

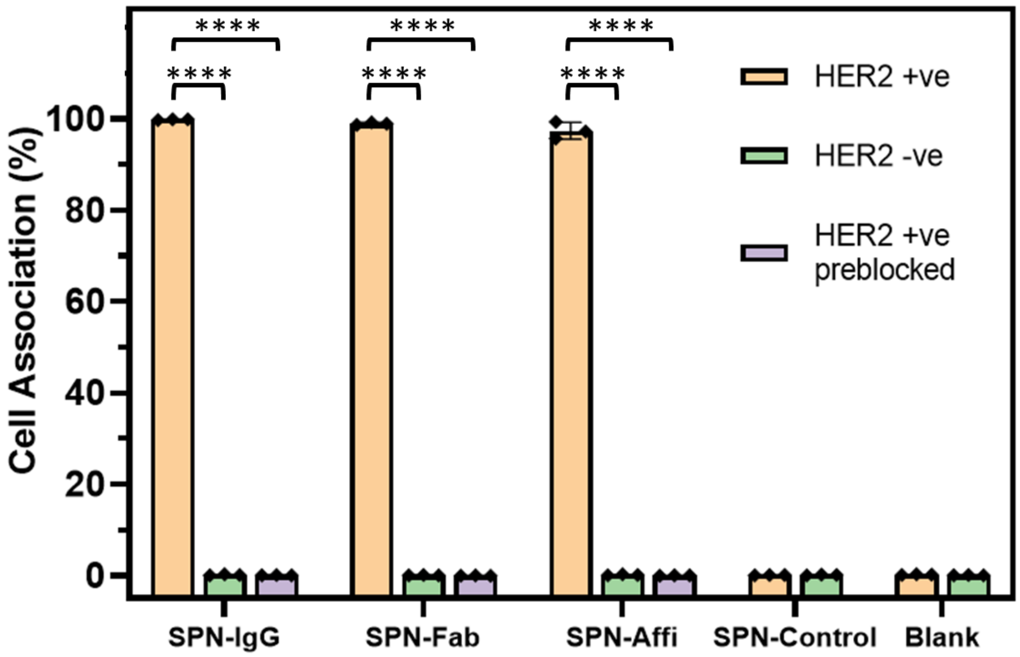

Figure S22. Cell association calculated from flow cytometry histograms (N = 1 for HER2 -ve and HER2 +ve preblocked, N = 3 for HER2 +ve, n=3 technical replicates for all). Statistical analysis: one-way ANOVA with Dunnett's multiple comparisons test, ****P < 0.0001.

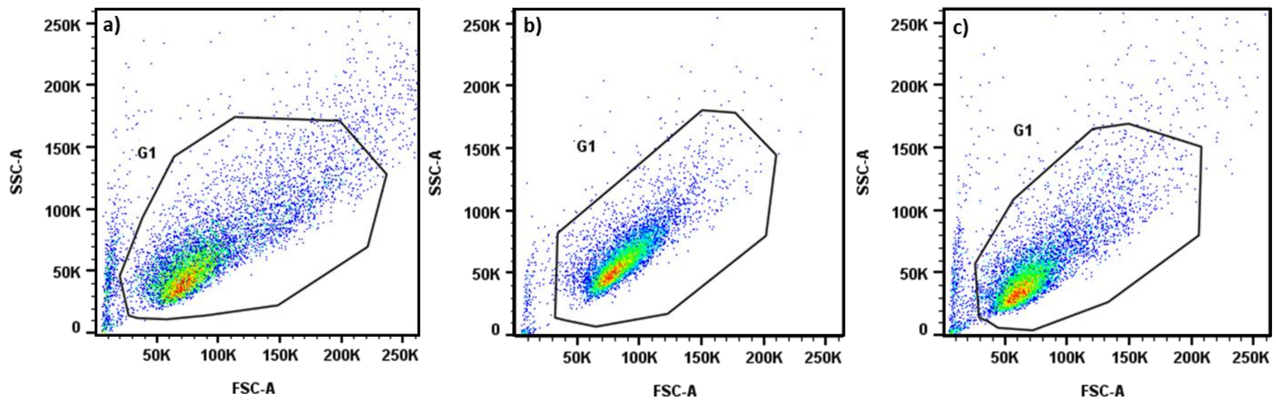

Figure S23. Representative dot plot of a) SKOV3 cells, b) MDA-MB-468 and c) SKOV3 pre-blocked with unconjugated IgG (SSC vs FSC).

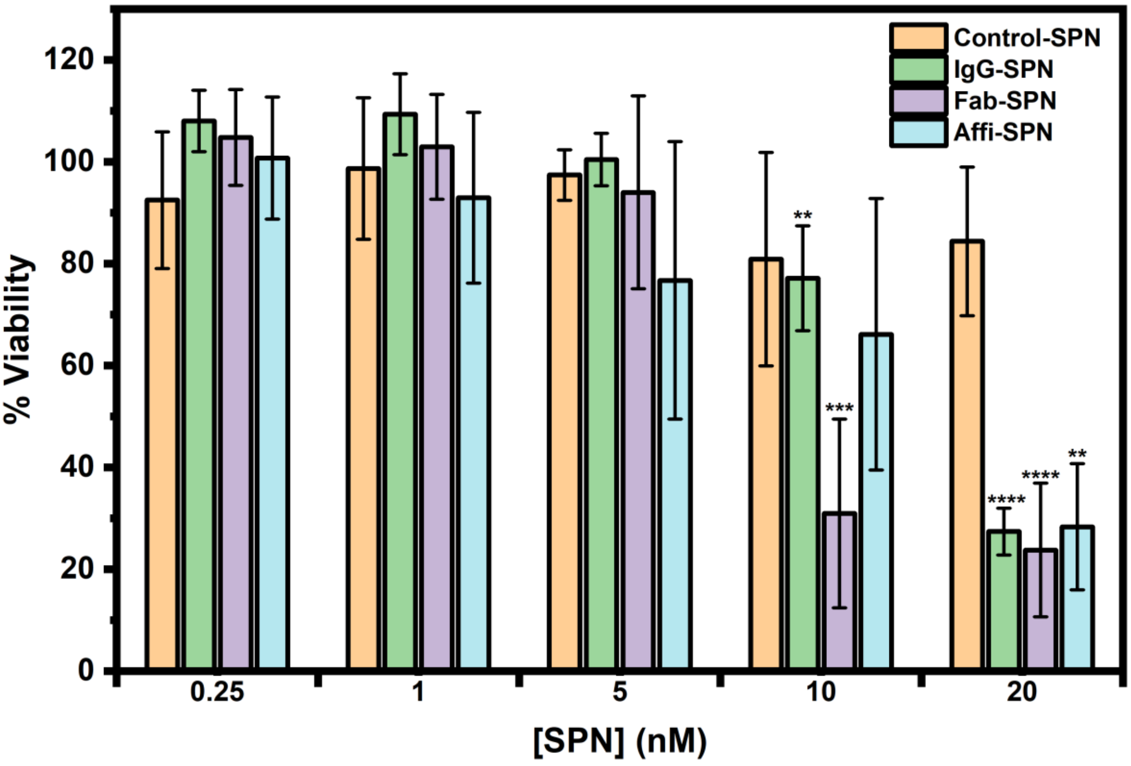

Figure S24. MTT assay of SPNs incubated with HEK-293 cells, 72 hours post-incubation. Data are presented as mean ± standard deviation (N = 3 biological replicates, n ≥ 2 technical replicates). Statistical analysis: one-way ANOVA with Dunnett's multiple comparisons test, comparison to PBS. Statistically significant results are indicated with asterisks: **P < 0.01, ***P < 0.001, ****P < 0.0001.

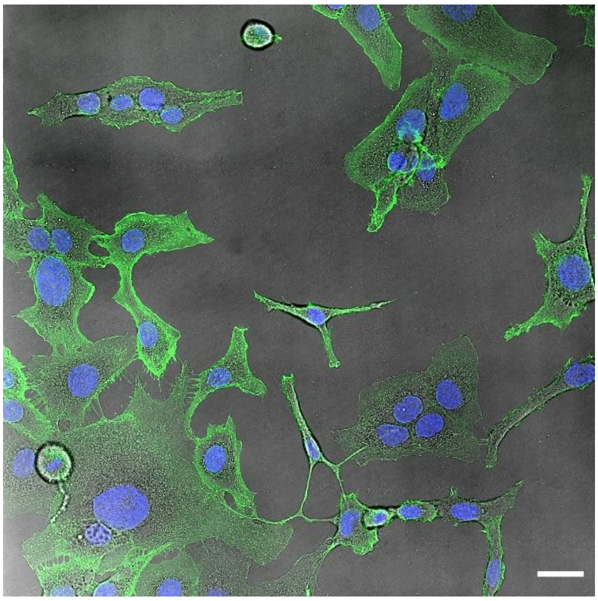

Figure S25. Further confocal image of SKOV3 cells labelled with DAPI (blue) and SPN-Affi (green) with overlayed brightfield (grey). Scale bar = 30 µm.

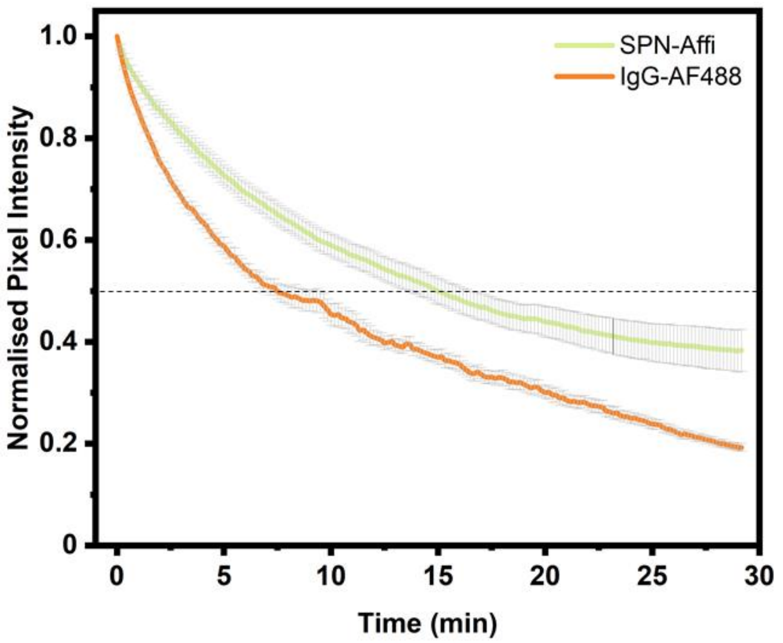

Figure S26. Photobleaching curves of SPN-Affi and IgG-AF488 labelled SKOV3 cells imaged every 10s under maximum laser power (488 nm). Dotted line indicates photobleaching half-life. Data are presented as mean ± standard deviation (n = 5 regions of interest).

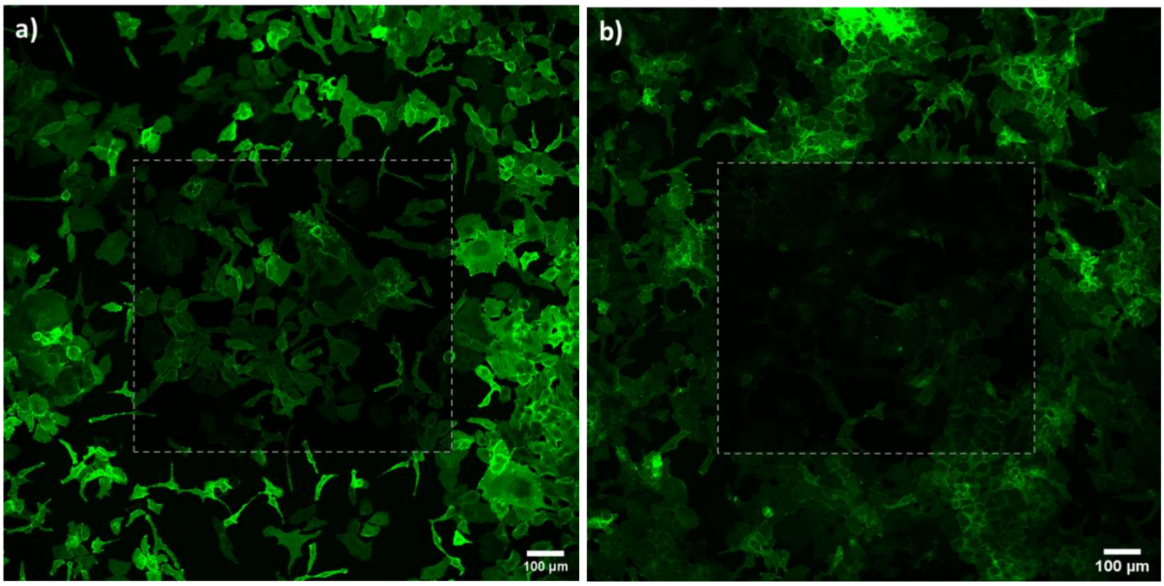

Figure S27. Confocal images of a) SPN-Affi and b) IgG-AF488 labelled SKOV3 cells after photobleaching. Dotted square indicates the area in which photobleaching was performed (30 min at maximum confocal laser power).

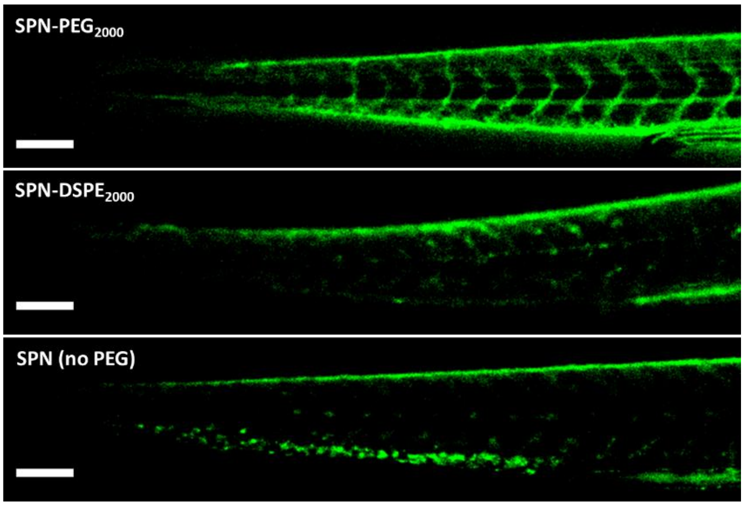

Figure S28. Representative stereomicroscope images of the tail region of zebrafish 24 hours post-injection of SPN-PEG_{2000}, SPN-$DSPE_{2000}$ and SPN (no PEG). Scale bar = 200 µm.

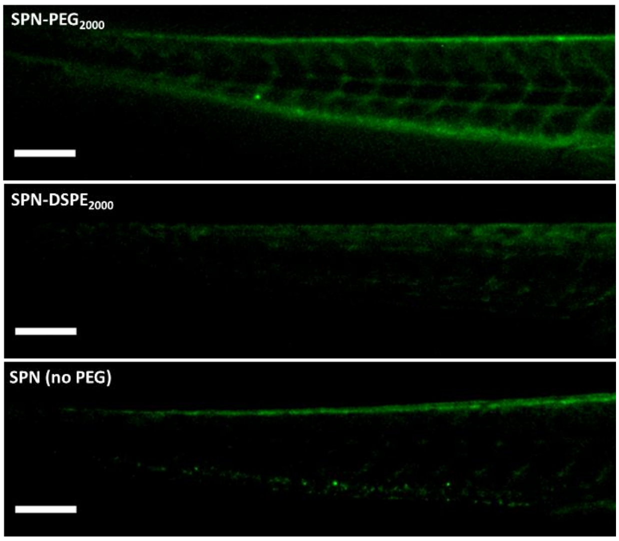

Figure S29. Representative stereomicroscope images of the tail region of zebrafish 48 hours post-injection of SPN-PEG$_{2000}$, SPN-DSPE$_{2000}$ and SPN (no PEG). Scale bar = 200 μm.

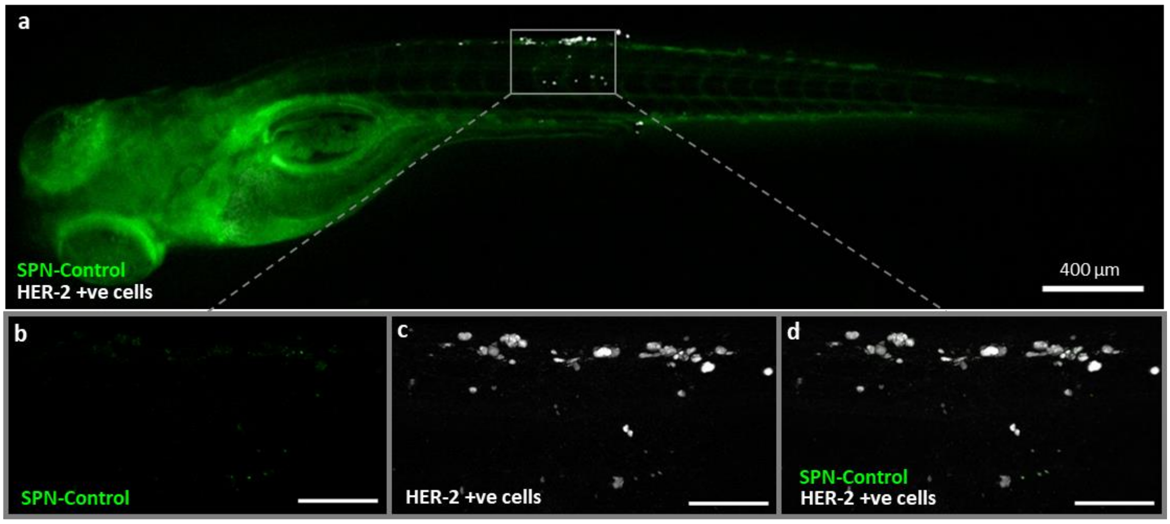

Figure S30. a) Representative confocal image of a zebrafish embryo xenograft 24 hours after SPN-Control injection. The solid box area is enlarged in b), c) and d) showing the fluorescent signal in the confocal stack for SPN-Control, cancer cells and SPN-Control/cancer cells combined, respectively (scale bar = 100 µm).

Supplementary Methods

See experimental methods section in main text for SPN fabrication, FCS measurements, protein-BCN synthesis, targeted SPN synthesis, flow cytometry, cytotoxicity and confocal imaging.

1. Equipment and reagents used

Equipment:

- UV-Vis absorption was measured using a Nanodrop 2000C spectrophotometer (Thermo Scientific).
- The majority of DLS data was obtained using a ZetaSizer Nano ZS (Malvern). Long term DLS data for PEG grafting density (Figure S15) was obtained using a Wyatt DynaPro Plate Reader III.
- LC-MS data was obtained with a 1260 Infinity (Agilent) using a C4 column for HPLC separation. Data was analysed using UniDec50.
- Fluorescent spectra were recorded on a SpectraMax M5 series (Molecular Devices) plate reader
- NMRs were recorded on either a Bruker AV-400 (400 MHz) or a Bruker Avance III HD (600 MHz) – equipment used is indicated for each spectra by the MHz number.
- Weight-average (Mw) and Number-average (Mn) molecular weights were determined with an Agilent-Technologies 1260 Infinity GPC System with 1260 RID. Measurements were performed at 40 °C, using analytical grade chloroform as eluent with two PLgel 10 µm MIXED B columns in series. Molar mass, as a function of elution time through the columns was calibrated using known molecular weight Agilent EasiVial narrow dispersity polystyrene standards. Samples were prepared using analytical grade chloroform in concentrations of ~1–2 mg/mL and filtered with VWR PTFE membrane 0.2 µm syringe filters before submission. An injection volume of 50 µL and GPC flow rate of 1.00 mL/min was used. GPC of graft-copolymers did not pass through the GPC column, so a molecular weight was not obtained.
- TEM: TEM grids (Agar Scientific, Ultra-thin carbon support on lacy carbon, AGS187-4) were first plasma cleaned for 1 minute before sample addition. 5 µL of SPN solution in water (1 mg/mL) was drop casted onto the grid and incubated for 5 minutes before being wicked away. The sample was then negatively stained with two drops of uranyl acetate (2 wt%, 0.45 µm filtered), the second being incubated for 1 minute before wicking away. Grids were dried overnight and imaged on a JEOL JEM-2100F microscope at 200 kV.
- Photographs were taken with an iPhone 11.

Reagents:

- All reagents were purchased from Merck (Sigma Aldrich) unless otherwise specified.
- Ontruzant full antibody was purchased from UCLH in its clinical formulation (Samsung Bioepis, lyophilised). Ontruzant Fab was prepared by a sequential enzymatic digest of the full antibody with pepsin and papain, following a literature procedure.[1]
- Dibromopyridazinedione-PEG-BCN and monobromopyridazinedione-PEG-BCN linkers (Figure S21) were synthesised based on previously reported methods.[2,3]
- Affibody $Z_{HER2:2395\text{-}Cys}$ was supplied by AffibodyAB, Sweden.

- HO-PEG2K-N_3 was purchased from Biopharma PEG.
- FBS was purchased from Gibco.
- LFIA strips with a printed polystreptavidin test line were provided from Global Access Diagnostics (formally known as Mologic).
- MDA-MB-468, SKOV3 and HEK-293 cell lines were purchased from ATCC.

2. Polymer Synthesis

F8BT-F synthesis

F8BT-F was synthesised following previously published protocol.[5] Mn of 88.5 kDa, Mw of 215 kDa, Mw/Mn (Đ) = 2.43.

F8BT-PEG$_{750}$ synthesis

F8BT-F (5 mg, 0.009 mmol), poly(ethylene glycol) methyl ether (Mn 750 Da, 7.5 mg, 0.01 mmol) and NaOH (crushed, 15 mg, 0.375 mmol) were added to a high pressure microwave reactor vial. The vial was sealed with a septum and degassed with argon, before anhydrous chlorobenzene (1.5 mL) and DMF (0.5 mL). The solution was heated at 130 °C for 1 h. After cooling the reaction was diluted into chloroform and washed three times with HCl (1M) then water (MilliQ). Organics were separated and dried with magnesium sulfate. Solvent was removed under reduced pressure and polymer was dissolved in the minimal THF required. Solution was then run through a size exclusion column (Biobeed SX-1 in THF). Polymer was dried and washed with hexane, to remove BHT stabiliser (present in THF), yielding a yellow solid **F8BT-PEG$_{750}$** (8 mg, 68%).

^{1}H NMR (400 MHz, CDCl3) δ 8.25 – 7.65 (m, 7H), 4.40 – 4.28 (br, 2H), 3.86 – 3.26 (m, 66H), 1.24 – 0.68 (m, 34H).

^{19}F NMR (400 MHz, CDCl3) – No peaks observed.

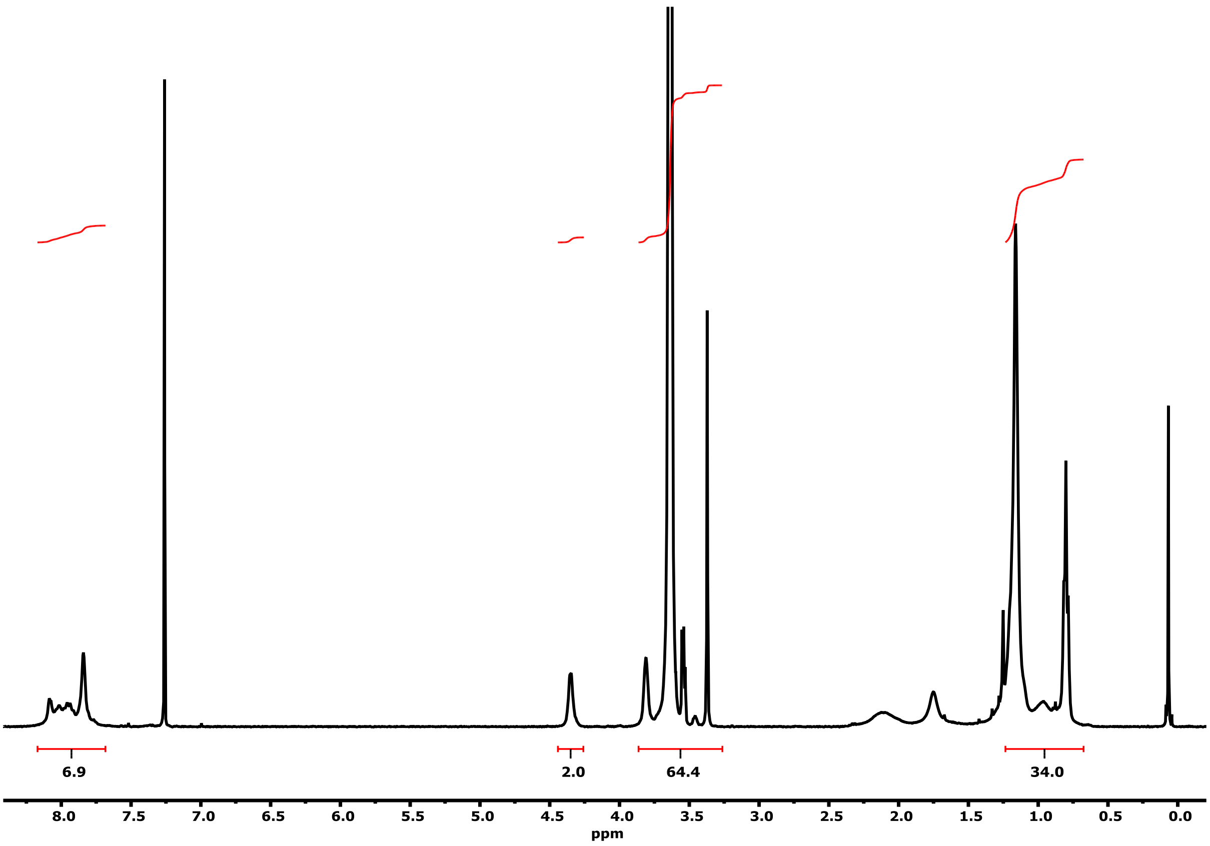

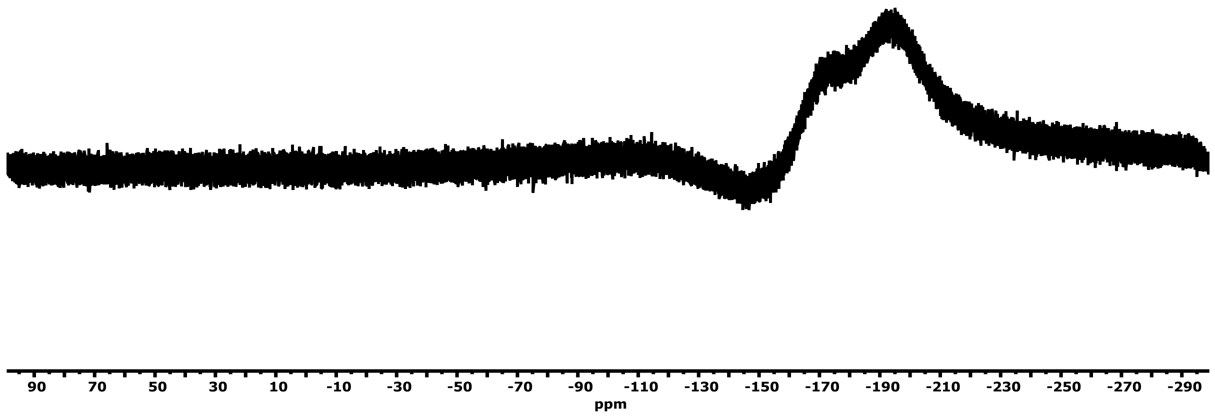

F8BT-PEG$_{2000}$ synthesis

F8BT-F (6 mg, 0.011 mmol), poly(ethylene glycol) methyl ether (Mn 2000 Da, 30 mg, 0.015 mmol) and KOH (crushed, 20 mg, 0.36 mmol) were added to a high pressure microwave reactor vial. The vial was sealed with a septum and degassed with argon, before anhydrous chlorobenzene (1.5 mL) and DMF (0.5 mL). The solution was to 120 °C for 2 h. After cooling, solvent was removed and MilliQ water (-7 mL) was added. Solid was stirred in water for 18 h. Water was discarded and replaced twice with MilliQ water. Polymer was then filtered (Nylon, 0.45 µm) and the polymer was dried overnight under reduced pressure, yielding a yellow solid **F8BT-PEG$_{2000}$** (11 mg, 40%).

^{1}H NMR (400 MHz, CDCl3) δ 8.27 – 7.64 (m, 7H), 4.45 – 4.22 (br, 2H), 3.98 – 3.31 (m, 184H), 1.24 – 0.72 (m, 34H).

^{19}F NMR (400 MHz, CDCl3) – No peaks observed.

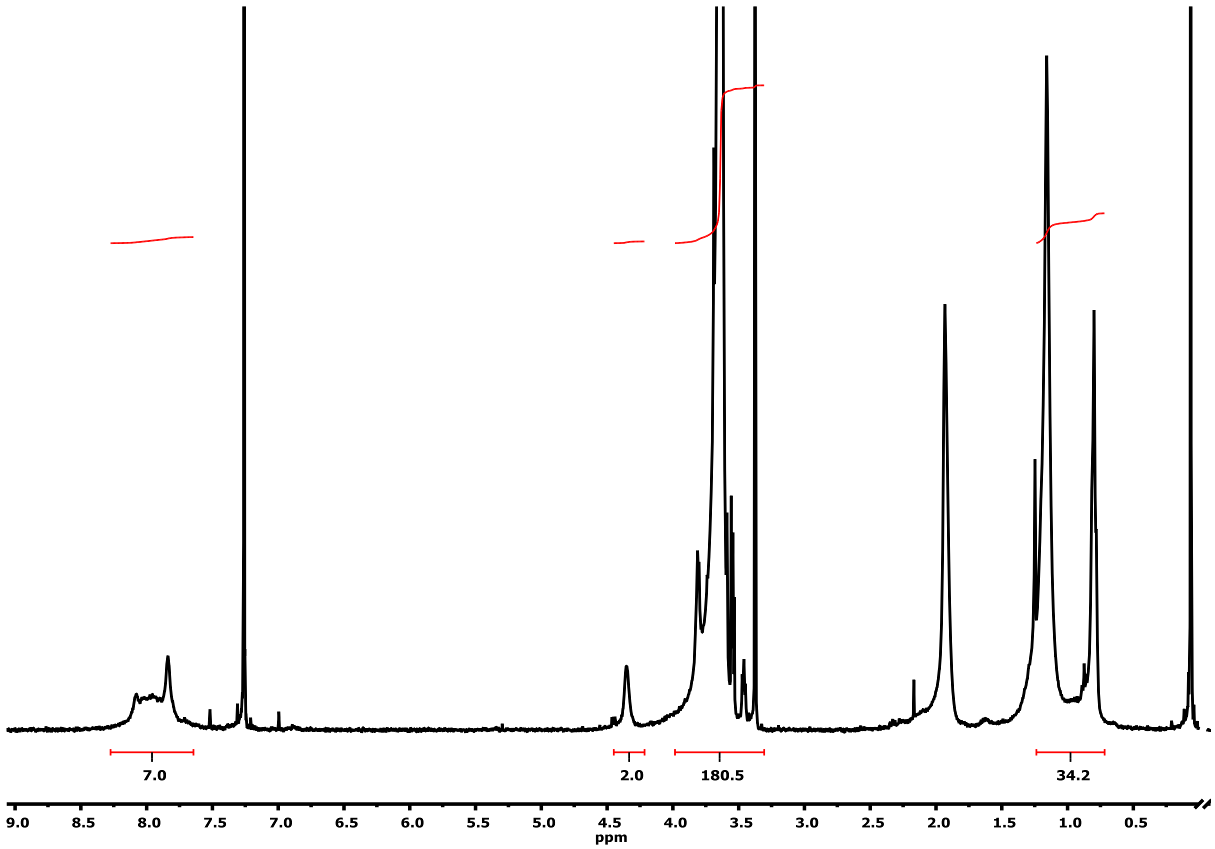

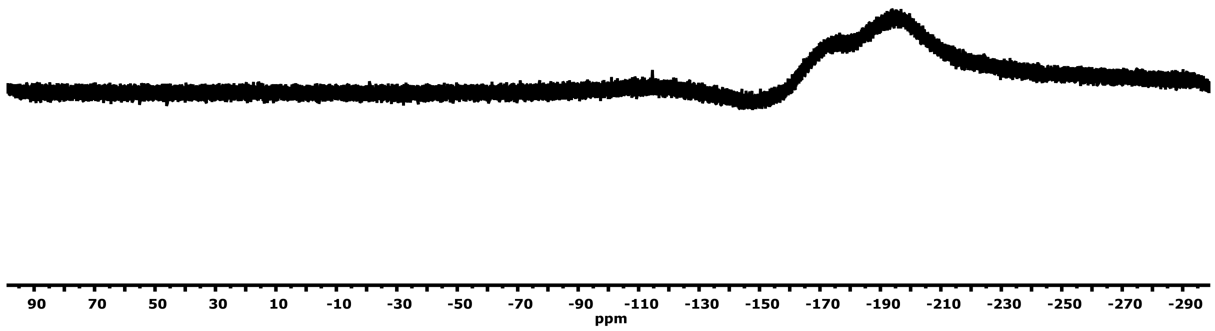

F8BT-PEG$_{5000}$ synthesis

F8BT-F (5 mg, 0.009 mmol), poly(ethylene glycol) methyl ether (Mn 5000 Da, 50 mg, 0.01 mmol) and KOH (crushed, 20 mg, 0.36 mmol) were added to a high pressure microwave reactor vial. The vial was sealed with a septum and degassed with argon, before anhydrous chlorobenzene (0.75 mL) and DMF (0.25 mL). The solution was to 100 °C for 1 h. After cooling, MilliQ water (~7 mL) was added and chloroform (1 mL) was added. Organic fraction was isolated and solvent removed under reduced pressure. Polymer was dissolved in acetone (1 mL) and added to a dialysis cassette (Float-A-Lyzer MINI, 20K MWCO) and placed into a beaker containing 4 L of MilliQ water. Polymer was removed from dialysis, water was removed by lyophilisation yielding a yellow solid **F8BT-PEG$_{5000}$** (11 mg, 22% yield).

In this case NMR was challenging due to the very large ratio of PEG protons to F8BT protons. Integrations from proton NMR were therefore not as accurate, especially for the 7 aromatic protons (δ

8.20 – 7.71). However, fluorine NMR exhibited no detectable peaks – suggesting that full substitution had occurred. Below is the predicted integrations listed, spectra shows the true integrations.

^{1}H NMR (400 MHz, CDCl3) δ 8.20 – 7.71 (m, 7H), 4.47 – 4.27 (br, 2H), 3.98 – 3.27 (m, 453H), 1.39 – 0.73 (m, 34H).

^{19}F NMR (400 MHz, CDCl3) – No peaks observed.

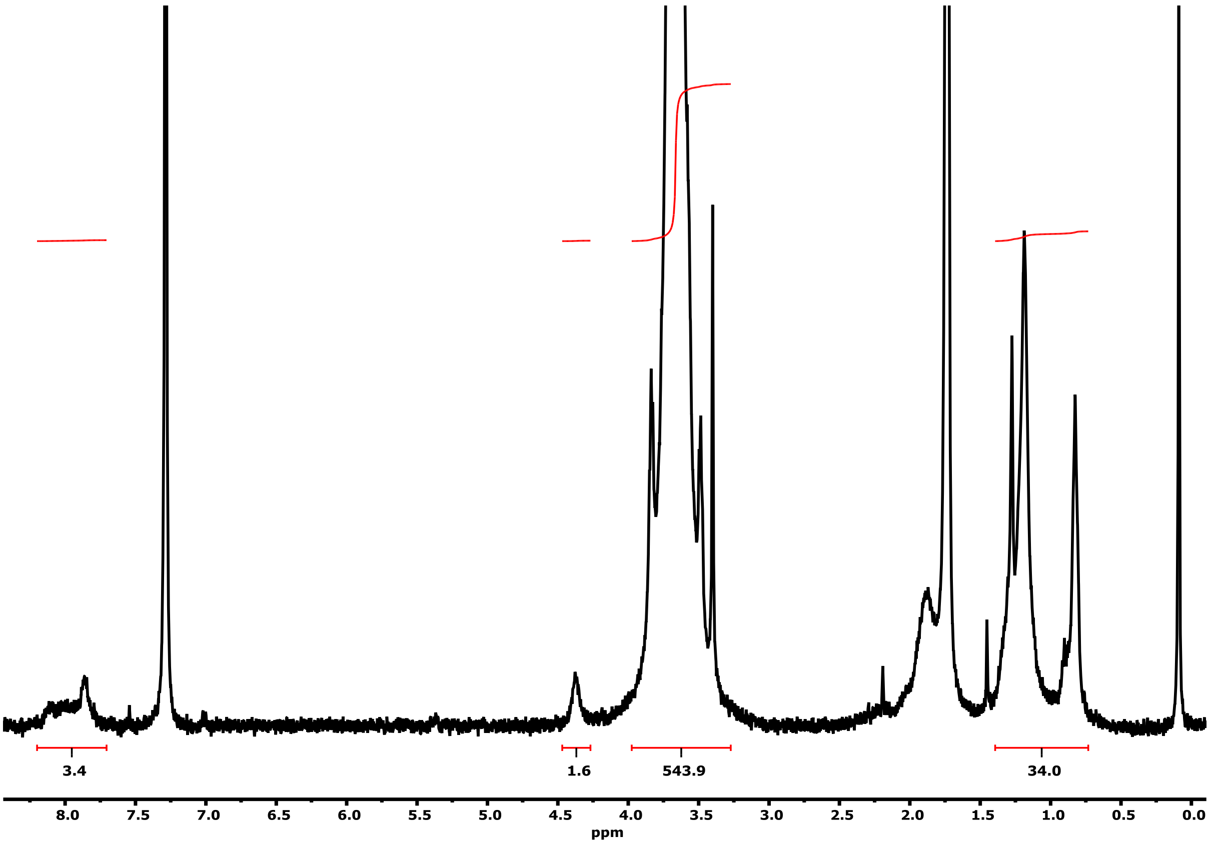

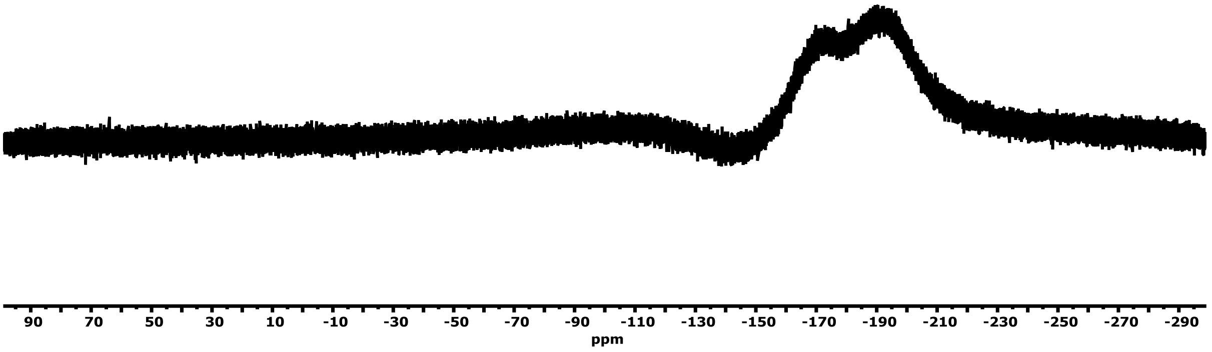

F8BT-PEG$_{2000}$-N$_3$ synthesis

F8BT-F (10 mg, 0.018 mmol) and NaOH (crushed, 40 mg, 0.74 mmol) were added to a high pressure microwave reactor vial. The vial was sealed with a septum and degassed with argon, before anhydrous chlorobenzene (1.5 mL) and DMF (0.5 mL). The solution was heated at 120 °C for 30 min. , Poly(ethylene glycol) azide (Mn 2000 Da, 19 mg, 0.009 mmol) was then added and the reaction was

heated for 1 h further. After cooling the solvent was removed under reduced pressure. The resulting yellow solid washed with water (30 mL) over a nylon filter. Polymer was extracted with THF and solvent was removed under reduced pressure. Resulting polymer was dried overnight for 18 h, yielding a yellow solid **F8BT-PEG$_{2000}$-N$_3$** (31 mg, 67%).

^{1}H NMR (400 MHz, CDCl3) δ 8.23 – 7.62 (m, 7H), 4.41 – 4.25 (br, 2H), 3.89 – 3.33 (m, 66H), 1.24 – 0.68 (m, 34H).

Note: peaks marked with an X below are BHT stabaliser from the THF solvent

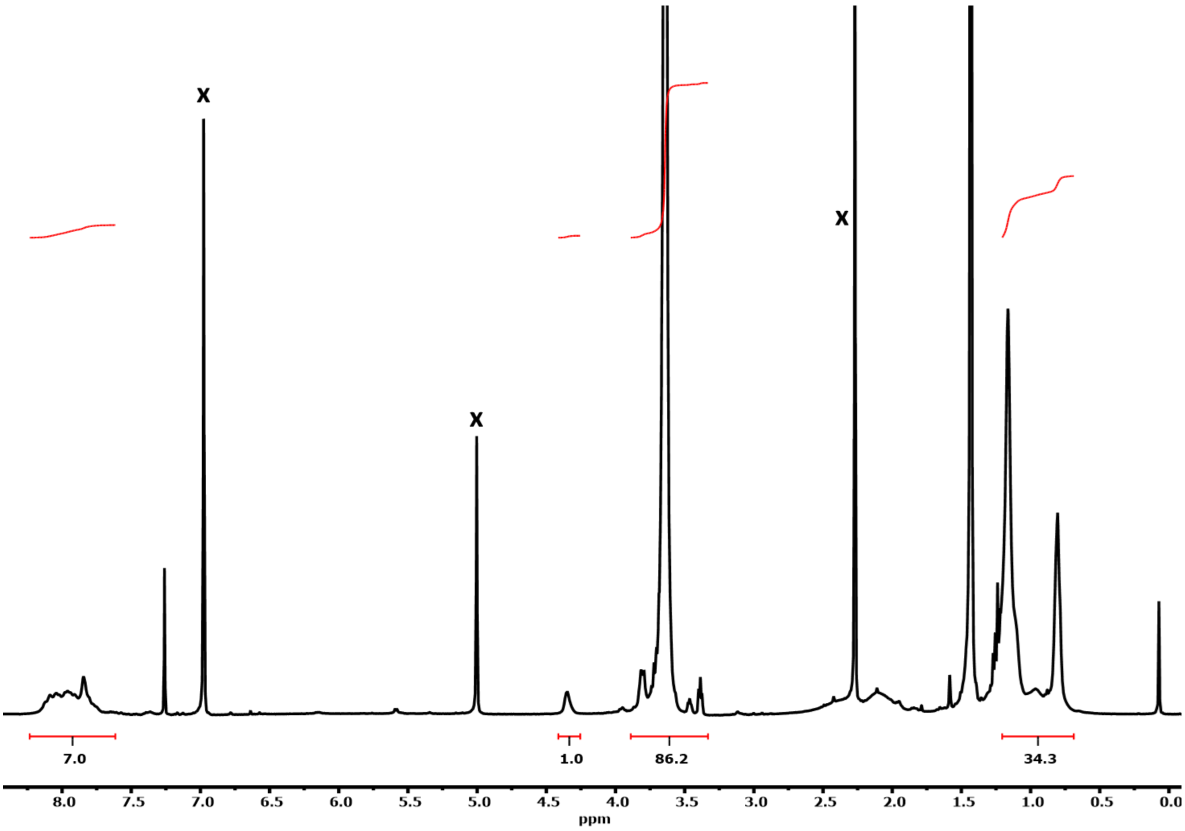

F8BT-PEG$_{2000}$ synthesis with varied grafting density

See below for amount of PEG added, yield and NMR characterisation for each in the series.

For this series – the synthetic method was altered to use a microwave, to improve the throughput.

F8BT-F (see table below), poly(ethylene glycol) methyl ether (Mn 2000 Da, see table below) and NaOH (crushed, 15 mg, 0.375 mmol) were added to a high pressure microwave reactor vial. The vial was sealed with a septum and degassed with argon, before anhydrous chlorobenzene (1.5 mL) and DMF (0.5 mL). The solution was heated in the microwave at 150 °C for 1 h. After cooling the reaction was diluted into chloroform and washed three times with HCl (1M) then water (MilliQ). Organics were separated and dried with magnesium sulfate. Solvent was removed under reduced pressure and polymer was dissolved in the minimal THF required. Solution was then run through a

size exclusion column (Biobeed SX-1 in THF). Polymer was dried and washed with hexane, to remove BHT stabiliser (present in THF), yielding a yellow solid (see table below).

Grafting density (%)	F8BT-F		PEG methyl ether		Yield	
	Mass (mg)	mmol	Mass (mg)	mmol	Mass (mg)	%
**100**	4.6	*0.008*	20	*0.010*	9	42
**75**	4.3	*0.008*	12.5	*0.0087*	4	25
**50**	5.5	*0.010*	10.6	*0.0084*	4.7	30
**25**	4.8	*0.009*	4.6	*0.0064*	3.9	43
**10**	4.4	*0.008*	1.6	*0.0044*	3.2	53

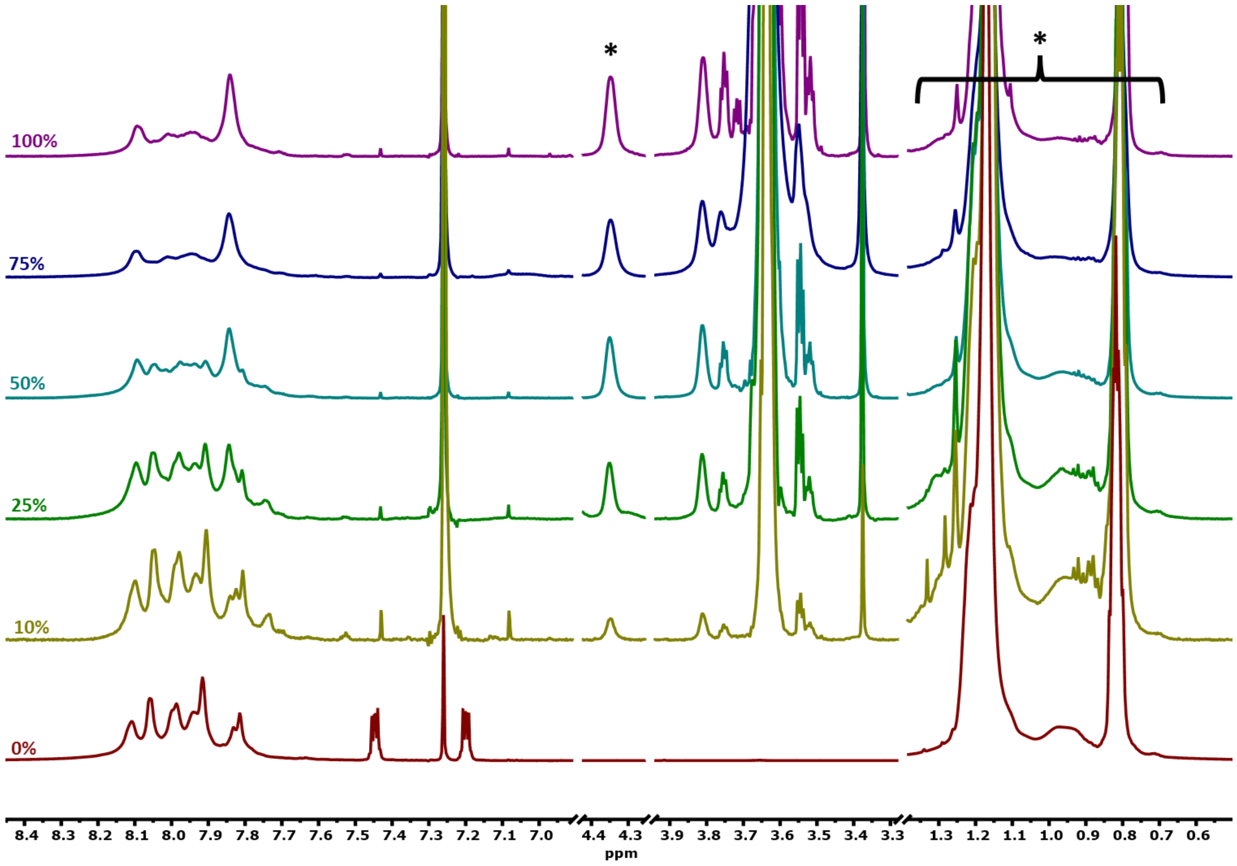

NMR: Stacked ^{1}H spectra of the PEG series (400 MHz, CDCl3). * indicates key peaks of which the integrations were used to estimate the PEG grafting density below.

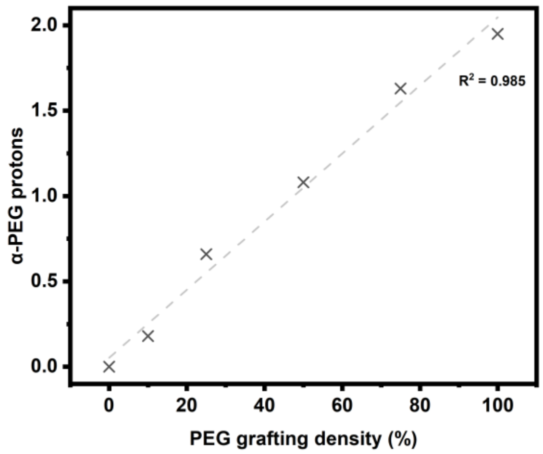

NMR. Plot of the integration of the α-PEG protons appended to the F8BT backbone (δ 4.41 – 4.29, see above) integrated with respect to the aliphatic F8BT side chains (δ 1.40 – 0.75, 34H, see above). Note this value should be equal to 2 when polymer is fully functionalised.

F8BT-PEG_{2000}-N_3 synthesis with varying azide percentage

See table below for amount of PEG added, yield and NMR characterisation for each in the series.

F8BT-F (5 mg, 0.009 mmol), poly(ethylene glycol) methyl ether (Mn 2000 Da, see below), poly(ethylene glycol) azide (Mn 2000 Da, see below) and NaOH (crushed, 15 mg, 0.375 mmol) were added to a high pressure microwave reactor vial. The vial was sealed with a septum and degassed with argon, before anhydrous chlorobenzene (1.5 mL) and DMF (0.5 mL). The solution was heated in the microwave at 130 °C for 2 h. After cooling the reaction was diluted into chloroform and washed three times with HCl (1M) then water (MilliQ). Organics were separated and dried with magnesium sulfate. Solvent was removed under reduced pressure and polymer was dissolved in the minimal THF required. Solution was then run through a size exclusion column (Biobeed SX-1 in THF). Polymer was dried and washed with hexane, to remove BHT stabiliser (present in THF), yielding a yellow solid (see below).

**Percentage azide content (%)**	**F8BT-F**		**PEG methyl ether**		**PEG azide**		**Yield**	
	**Mass (mg)**	**mmol**	**Mass (mg)**	**mmol**	**Mass (mg)**	**mmol**	**Mass (mg)**	**%**
**1**	5.3	*0.010*	19.3	*0.0097*	0.2	*0.0001*	15.5	63
**5**	4.9	*0.009*	17.3	*0.0087*	0.9	*0.0005*	16.8	74
**10**	5	*0.009*	16.7	*0.0084*	1.9	*0.0010*	15.3	66
**25**	4.6	*0.008*	12.8	*0.0064*	4.4	*0.0022*	11.5	54
**50**	4.8	*0.009*	8.8	*0.0044*	8.9	*0.0045*	8	36
**100**	4.9	*0.009*	0	*0.0000*	18	*0.0090*	6.1	27

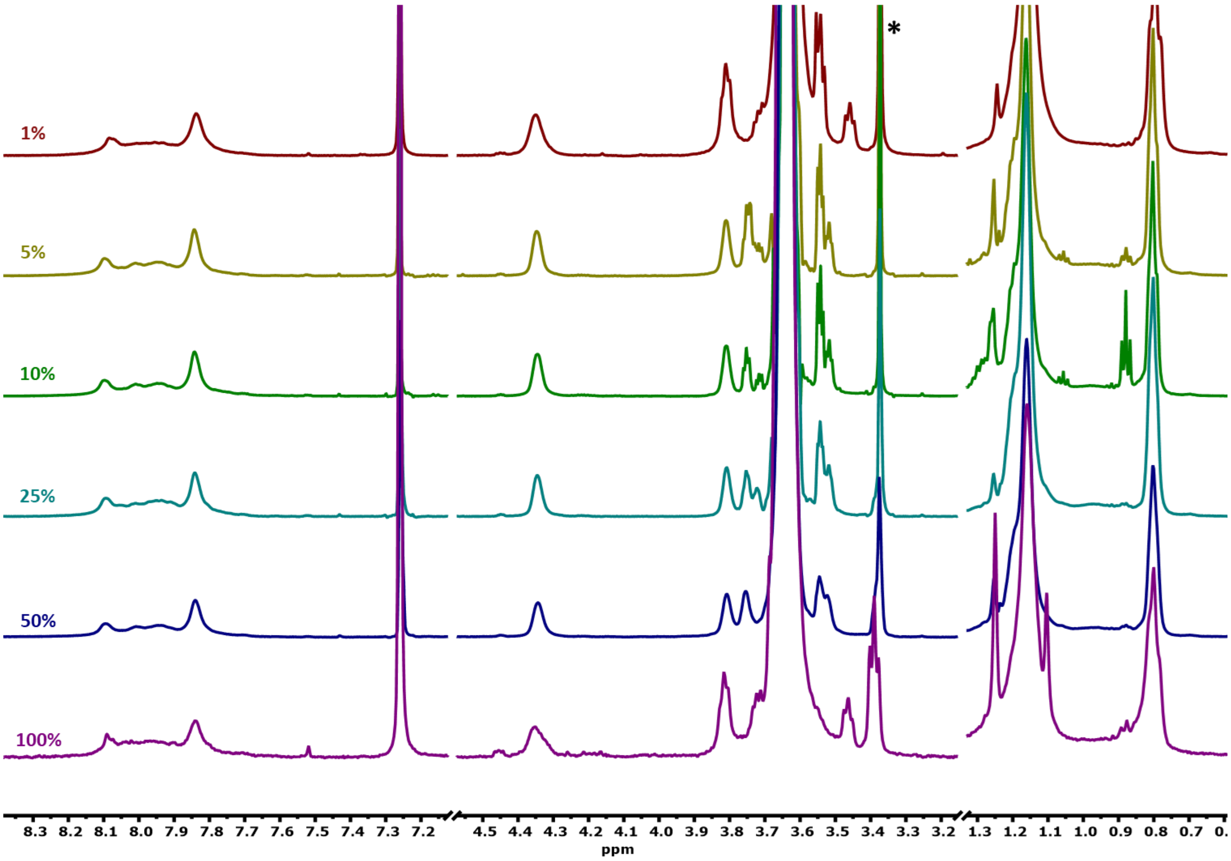

NMR: Stacked ^{1}H spectra of the azide series (400 MHz, CDCl3). * indicates peak which is focused below.

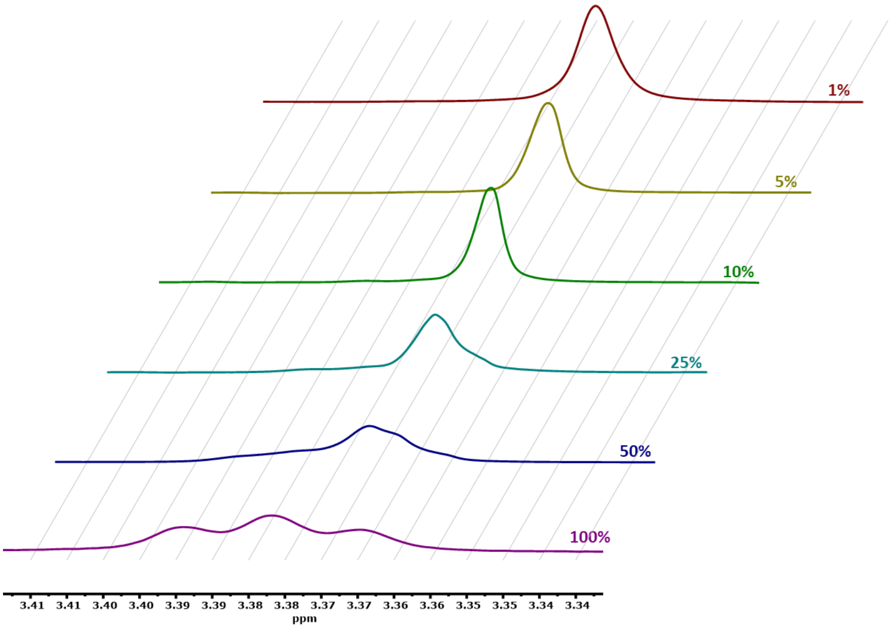

NMR: Stacked ^{1}H spectra of the azide series(400 MHz, CDCl3) focused on the terminal protons on the PEG chain. Note the gradual change in peak shape due to the ratio of methyl ether (-O**<u>CH</u>**$_3$, singlet) to azide (CH_2-**<u>CH</u>**$_2$-N_3, triplet).

3. Colloidal Stability of SPNs

Colloidal stability of SPN-PEG$_{750}$, SPN-PEG$_{2000}$ and SPN-PEG$_{5000}$.

Solutions of SPN-PEG$_{750}$, SPN-PEG$_{2000}$ and SPN-PEG$_{5000}$ were prepared via the nanoprecipitation method. Each solution was diluted ten-fold into water and PBS and left at room temperature for 24 h. The process was repeated with the addition of FBS (final concentration of 10 v/v%) and the resulting solutions were incubated at 37 °C for 24 h. The particles were then analysed by FCS and DLS.

Colloidal stability of Lipid-coated SPNs and PEG blend.

Lipid: SPN-DSPE$_{2000}$ were prepared via a modified nanoprecipitation method where the initial THF solution also contained lipid. Each solution was diluted ten-fold into water and PBS and left at room temperature for 24 h. The process was repeated with the addition of FBS (final concentration of 10 v/v%) and the resulting solutions were incubated at 37 °C for 24 h. The particles were then analysed by FCS and DLS.

PEG blend: Separate solutions of F8BT-F and poly(ethylene glycol) methyl ether (Mn = 750, 2000 and 5000) were prepared in THF (1 mg/mL). The following mixtures were prepared to emulate the F8BT-F PEG ratio of the graft polymer.

- PEG-750. 420 µL F8BT-F + 580 µL PEG-750.
- PEG-2000. 210 µL F8BT-F + 790 µL PEG-2000.
- PEG-5000. 100 µL F8BT-F + 900 µL PEG-5000.

Solutions were then used following the nanoprecipitation method to yield nanoparticle solutions in water. Each solution was diluted ten-fold into water and PBS and left at room temperature for 8 h. The particles were then analysed by FCS.

Comparing graft-copolymer to two-component approach

SPN prep: SPN-DSPE$_{2000}$-N$_3$ and SPN-PEG2k-N$_3$ was prepared by following the nanoprecipitation method. The UV-Vis spectra was then recorded of the SPN solutions and the concentration of DSPE SPNs was adjusted to match (so that the semiconducting polymer content was the same).

Cy5 functionalisation: DBCO-cyanine 5 (500 µM in DMSO, 200 µL) was added to SPN solution (200 µL). Reactions were left on an orbital shaker overnight at room temperature. The solutions were then purified using centrifuge filters (Amicon 0.5 mL, 100KDa MWCO) five times with milliQ water to remove excess dye. The resulting nanoparticles were then filtered through a PVDF filter (0.45 µM) and the particles were then matched in concentration as before.

Stability study: In PCR tubes, SPN solutions (10 µL) were diluted ten-fold into PBS and FBS then incubated at 37 °C. The following day this process was repeated. The following day the process was repeated once more prior to measurement via FCS.

Colloidal stability of SPN-PEG grafting series.

SPNs were generated from their corresponding polymer following the nanoprecipitation method into water (MilliQ). SPNs were then diluted two-fold into PBS (2X) and water. DLS was then recorded

after one day, one week and one month. The SPNs were also diluted into FBS as above and the series was also studied by FCS after initial dilution, 8 hours and then 24 hours later.

Colloidal stability of SPN-PEG$_{2000}$-N$_3$ in buffer solutions

SPN-PEG$_{2000}$-N$_3$ nanoparticles were generated following the nanoprecipitation method into water (MilliQ). SPNs were diluted two-fold into the following buffer solutions: MES (pH 5.4, 100 mM), carbonate (pH 9.8, 200 mM), HEPES (pH 7.4, 200 mM), BBS (pH 8, 100 mM), PBS (ph 7.4, 2X) and MilliQ water. Resulting SPN solutions were left at room temperature for 4 days and were then analysed by FCS.

4. Azide functionalisation study

Azide functionalisation with FRET acceptor

SPNs were generated from their corresponding polymer, with varied azide percentage content, following the nanoprecipitation method into water. Each SPN solution (50 µL) was added DBCO-AF594 (1 mM in DMSO, 1 µL) and left on an orbital overnight at room temperature to fully react. The solutions were then purified using centrifuge filters (Amicon 0.5 mL, 100KDa MWCO) to remove excess dye; Nanoparticles were added above the filter membrane and the tubes were centrifuged (12,000 rcf, 3 min). Concentrated nanoparticles were diluted to the original volume with water and the filtrate was discarded. The process was repeated a further four times. Particles were purified further by passing solution through a desalting centrifuge column (ZebaSpin 0.5 mL, 40K MWCO, 1500 rcf, 2 min). The resulting nanoparticles were then filtered through a PVDF filter (0.45 µM). Fluorescent spectra were recorded with excitation at 450 nm and images were taken under UV light exposure in a dark room.

5. SPN functionalisation with biorecognition elements

Affi-BCN characterisation:

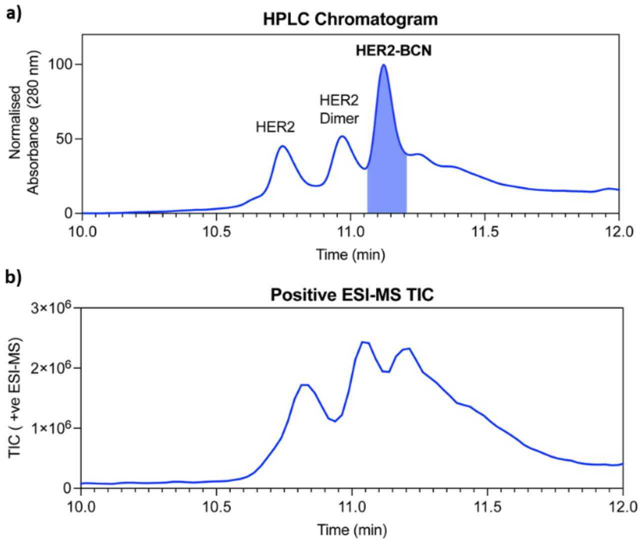

LC-MS data. Affi-BCN conjugate. a) HPLC chromatogram at 280 nm. b) Total Ion Count (TIC) for the positive mode ESI-MS signal.

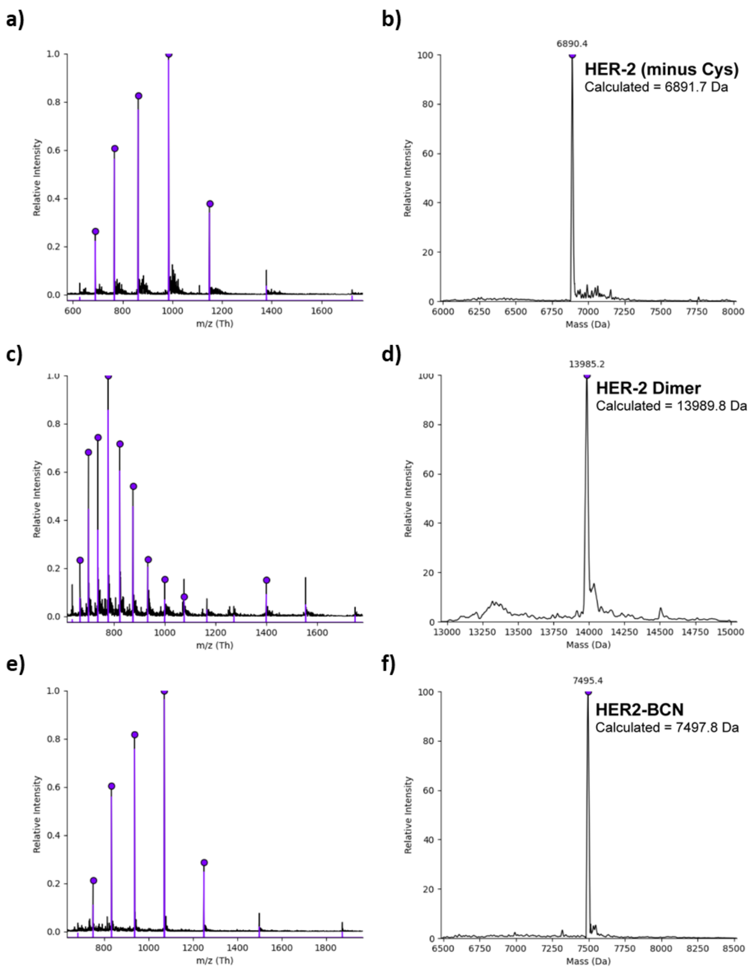

LC-MS deconvolution. Affi-BCN LC-MS data was deconvoluted using UniDec 5.05. a) Extracted positive mode ESI-MS data (t = 10.764 to 10.913 min) with deconvolution showing the fit charged states and b) the mass obtained from deconvolution which fits to affibody minus a cysteine fragmentation product. c) Extracted positive mode ESI-MS data (t = 10.988 to 11.113 min) with deconvolution showing the fit charged states and d) the mass obtained from deconvolution which fits to an affibody disulphide bridged dimer. e) Extracted positive mode ESI-MS data (t = 11.162 to 11.237 min) with deconvolution showing the fit charged states and f) the mass obtained from deconvolution which fits to affibody modified with the PD-BCN linker.

SPN-PEG$_{2000}$-N$_3$ concentration estimation

Prior to SPN functionalisation, an estimation of particle concentration was recorded using FCS. This was correlated to an absorption by UV-Vis so that particle concentration could be estimated readily. SPN-PEG$_{2000}$-N$_3$ was found to have a concentration of 31.4 ± 7.9 nM (A_{450} = 0.588, 1 cm pathlength).

6. HER2 targeting

LFIA

In a 96 microwell plate SPN-IgG, SPN-Fab and SPN-Affi (2 µL) were diluted into FBST (FBS with 0.05 v/v% of TWEEN20, 20 µL) in duplicate. To each pair of wells (for SPN-IgG, Fab and Affi), HER-2 biotin (20 nM in PBST, 20 µL) and just PBST (20 µL) was added. Nitrocellulose strips with a printed polystreptavidin test line (Mologic) were added to each well and the solution was allowed to wick up the strip until dry. Strips were then imaged under UV light.

FLISA

See below for plate design for this experiment:

	**1**	**2**	**3**	**4**	**5**	**6**	**7**	**8**	**9**	**10**	**11**	**12**
**A**	SPN-Affi			SPN-Fab			SPN-IgG				SPN-Control	
**B**	SPN-Affi			SPN-Fab			SPN-IgG					
**C**	SPN-Affi			SPN-Fab			SPN-IgG				SPN-Control	

= HER2 biotin coated

= HER2 biotin + blocking protein

= Blank

Streptavidin coating: To a 96-well microplate (Greiner half-area, black, high-binding surface) was added streptavidin solution (20 µL, 1 µg/mL in 50 mM carbonate buffer pH 9.8) in each well. Plates were kept at 4 °C for 18 h. Plate were then washed by removing the solution and adding PBST (100 µL), removing PBST and repeating this step twice more.

HER2 biotin coating: To the appropriate wells (row A and B, see above), HER2 biotin (20 µL, 100 nM in PBST) was added. To 'blank' wells was added PBST (20 µL). Plates were kept at room temperature for 30 min. All wells were then washed three times with PBST as before, wells were kept in PBST (100 µL) after final wash.

Blocking: PBST was then removed from the wells which required blocking (row B). Then affibody solution (20 µL, 100 nM in PBST) was added to wells for SPN-Affi incubation (B1-B3). Ontruzant (20 µL 100 nM in PBST) was added to those wells for SPN-Fab and SPN-IgG incubation (B4-B9). The remaining wells were kept in PBST and all were left at room temperature for 30 min. All wells were subsequently washed with PBST three times.

Particle incubation: SPN-PEG$_{2000}$-N$_3$ (SPN-Control), SPN-Affi, SPN-Fab and SPN-IgG (20 µL, 1 nM in PBST) were added to the appropriate wells. Plates were kept at room temperature for 1 h. All wells were then washed three times with PBST as before.

Fluorescence spectra of each well was recorded in a plate reader (Ex 450 nm, Em 480-600 nm).

7. Photostability

IgG-AF488 synthesis (for photostability study)

In a microtube (0.5 mL Eppendorf Protein LoBind), IgG-BCN (10 µL, 40.9 µM in carbonate buffer (pH 9.8, 100 mM)) was added AlexaFluor-488 azide (0.8 µL, 10 mM in DMSO). The solution was left to react on an orbital shaker at room temperature for 18 h. Excess dye was removed via a desalting column (ZebaSpin 0.5 mL 40K MWCO) resulting in a yellow solution (2.8 µM, 80 µL, yield of 55%).

Concentration of IgG was estimated by using the extinction coefficient of AF488 (73,000 $M^{-1}cm^{-1}$ at A_{495}) to obtain a dye concentration. Then on the assumption of four dyes per IgG the IgG concentration was estimated.

Confocal imaging and analysis

SKOV3 cells were stained following the same protocol for confocal microscopy with DAPI and IgG-AF488 (100 nM in PBS). SPN-Affi and IgG-AF488 stained cells were then compared under accelerated photobleaching. Under a 20X objective, an area of interest was selected under 488 nm excitation with a laser power of 15%. The laser power was then set to 100% (80% argon). The gain was quickly adjusted to be below saturation. An image was then taken every 10s for 30 min (512x512 resolution, 8 reads per line). The laser power was then returned to the original and the objective was then decreased to 10X to take a final image (in both DAPI and 488 channel) with the photobleached region in the centre.

The photostability was analysed by choosing five fixed regions of interest (ROIs) for both images. The mean pixel intensity of those ROIs was plotted as a function of laser exposure time in Fiji.

8. Zebrafish circulation

Zebrafish maintenance and care

All experiments were performed in accordance with UK Home Office requirements (Animals Scientific Procedures Act 1986, project license P5D71E9B0). The transparent TraNac mutant fish were obtained from Julian Lewis, London Research Institute, London. Fish were kept at 28.5°C on a 14-hour light/10-hour dark cycle. CBS facility of Imperial College London and maintained according to standard practices. All embryos were raised in E2 water (with added 0.3 ppm methylene blue) at a density of ~50 embryos per Petri dish. The water was replaced daily.

SPN injections

Live zebrafish embryos were anesthetised in a solution of 4.2 w/v% MS-222 and mounted on a 2 w/v% agarose plate, 3 days post-fertilisation. Borosilicate injection capillaries were fabricated using a Flaming/Brown P-97 micropipette-puller (Sutter, Novato, CA, USA) with the settings: heat 855, pull 150, velocity 80 and time 94 from borosilicate capillaries (outer diameter 1.0 mm, inner diameter 0.78 mm, length 100 mm; Harvard, Apparatus, Holliston, MA, USA). Using the injection capillaries, SPN solution (0.5 nL) was then injected into the caudal vein of the embryos. The injections were performed using a Narishige IM300 microinjection pressure controller (Narishige-group Tokyo, Japan). An eyepiece reticule (NE120, Pyser-SGI, Edenbridge, UK) was used to monitor injection volume and the needle was controlled using a micromanipulator (M3301 Micromanipulator Right hand World Precision Instruments Ltd. Hitchin, UK) and stereomicroscope (Nikon SMZ-1000).

Zebrafish imaging

SPN circulation was recorded with a Leica Stereomicroscope (Leica M165 C) with 2.0x objective (Leica), and a Leica EL6000 external light source and Leica DFC7000 T camera. 10 s videos were captured using a GFP filter, 2x2 binning, 10x gain and 8x magnification.

Circulation efficiency calculations

Nanoparticle circulation time was quantified using custom Fiji macros (Supplementary File 1). SPN circulation was analysed within a region of interest (1200 x 450 µm) covering the tail region of the zebrafish embryos. The circulating SPNs were isolated by detecting only fluorescent signal that changed between frames. This was achieved by subtracting each individual frame of the video by its subsequent frame. This image series was then projected into a single image (maximum projection). The fluorescent pixel area in the maximum projection was then detected using a Fiji watershed plug in (Watershed on grey level images (http://bigwww.epfl.ch/) 02.2008 Biomedical Imaging Group (BIG), EPFL Lausanne, Switzerland.) with the following settings: Gaussian Blur radius, 2 pixels; 4-connected, Min/Max 0-80. The total area was then measured in Fiji using the built-in 'analyze particle' plug-in.

9. Zebrafish xenograft *In-vivo* targeting

Zebrafish maintenance and care

Experiments have been agreed with Mattilsynet, the Norwegian food safety authority (license FOTS id: 13563). After fertilization zebrafish embryos were placed in Petri dishes containing zebrafish egg water supplemented with 0.003 w/v% phenythiourea (PTU). Fish were kept in an incubator at 28.5 ˚C. AB WT zebrafish strains were used for this study.

Cancer cell xenotransplantation

The SKBR-3 line has been received as a gift from the group of Gunhild Mælandsmo at Radium University Hospital, Oslo. SKBR-3 had been cultured in RPMI medium supplemented with FBS (10 v/v%). Prior to xenotransplantation, the cells were incubated with CellMask^{TM} Deep red Plasma Membrane Stain (Invitrogen) at a concentration of 5 µg/ml for 30 min. They were then detached using Versene (Life technologies) and pelleted at 400 RCF. After removal of the supernatant, the cell pellet was used to load borosilicate injection capillaries for injections.

Zebrafish injections

In order to inject zebrafish embryos, borosilicate injection capillaries (GC100T-10 Harvard Instruments) were made using a needle puller (P-97, Sutter Instruments). A micromanipulator (Narishige MN-153) connected to an Eppendorf Femtojet Express pump was used to direct the needles for careful injection in the area of interest. Prior to injection, zebrafish were sedated (Finquel 0.02 w/v% in embryo water) and laid on a petri dish with a 2 w/v% agarose base (in milliQ water).

Cancer cell and nanoparticle injections

~150 SKBR-3 cells were injected in the neural tube of zebrafish embryo 3 day old as described in previous work.[6] Following xenotransplantation, the zebrafish were maintained at a temperature of 32 ˚C.

SPN-Affi and SPN-PEG$_{2000}$-N$_3$ (SPN-Control) (5 nL at 17 nM in PBST) were injected in the zebrafish caudal vein 24 hours after cancer cells xenotransplantation. One day after injection, zebrafish embryos were imaged on a Leica Stereomicroscope or with an Andor Dragonfly spinning disc confocal microscope.

Imaging and image analysis

Stereomicroscope: A Leica DFC365FX connected to fluorescence light and mounting a planapo 1.0 X lens was used for imaging of zebrafish at a magnification of 30X. At this magnification the whole animal can be imaged. For this type of imaging, zebrafish embryos had been sedated and placed on 2 w/v% agarose plates, as described above.

Spinning disc confocal: Zebrafish embryos were first sedated and then placed in a dish with glass bottom (MatTek) and covered in low melting point agarose. Once hardened, embryo water containing sedative was added on top (Finquel 0.02 w/v% in embryo water). Zebrafish were imaged with a 10X/0,45 or 20X/0.75 dry lenses. Image acquisition and stitching was made with the software Fusion. Image quantification was performed with ImageJ software and IMARIS. For imaging of cancer cells a 3D stack was taken for each tumor.

Image analysis: In order to quantify the targeting capabilities of SPN-Affi we injected zebrafish embryos with either SPN-Affi, SPN-Control, or PBS. Our goal was then to quantify the fluorescence of SPN (488 nm excitation) which arrived at the xenotransplanted cancer cells. Fluorescence of cancer cells in the green channel in PBS-injected zebrafish provided the background to be subtracted to our measurements of fluorescence of cancer cells in SPN-Affi and SPN-Control injected zebrafish. Analysis of SPN fluorescence in cancer cells in 3D stacks was made using IMARIS. The obtained value was then normalized for total zebrafish fluorescence with the values obtained imaging zebrafish at 30X with the stereomicroscope and quantifying the single plane image with Fiji.

The used formulas are as follow.

NP with affibodies score (arbitrary units):

$$\frac{(Fluorescence\ Affi - Fluorescence\ PBS)}{(Total\ Affi - Total\ PBS)} X1000000$$

NP control score (arbitrary units):

$$\frac{(Fluorescence\ control - Fluorescence\ PBS)}{(Total\ control - Total\ PBS)} X1000000$$

Legend

Cancer cell fluorescence in zebrafish injected with SPN-Affi: Fluorescence Affi
Cancer cell fluorescence in zebrafish injected with SPN-Control: Fluorescence control
Cancer cell fluorescence in zebrafish injected with PBS: Fluorescence PBS
Total fluorescence in zebrafish injected with SPN-Affi: Total Affi
Total fluorescence in zebrafish injected with SPN-Control: Total control
Total fluorescence in zebrafish injected with PBS: Total PBS

10. References

[1] D. A. Richards, A. Maruani, V. Chudasama, *Chem. Sci.* **2016**, *8*, 63.

[2] C. Bahou, D. A. Richards, A. Maruani, E. A. Love, F. Javaid, S. Caddick, J. R. Baker, V. Chudasama, *Org. Biomol. Chem.* **2018**, *16*, 1359.

[3] J. C. F. Nogueira, M. K. Greene, D. A. Richards, A. O. Furby, J. Steven, A. Porter, C. Barelle, C. J. Scott, V. Chudasama, *Chem. Commun.* **2019**, *55*, 7671.

[4] J. Mączyńska, C. Da Pieve, T. A. Burley, F. Raes, A. Shah, J. Saczko, K. J. Harrington, G. Kramer-Marek, *Cell Death Dis.* **2020**, *11*, 886.

[5] A. Creamer, C. S. Wood, P. D. Howes, A. Casey, S. Cong, A. V. Marsh, R. Godin, J. Panidi, T. D. Anthopoulos, C. H. Burgess, T. Wu, Z. Fei, I. Hamilton, M. A. McLachlan, M. M. Stevens, M. Heeney, *Nat. Commun.* **2018**, *9*, 3237.

[6] A. Kocere, J. Resseguier, J. Wohlmann, F. M. Skjeldal, S. Khan, M. Speth, N. J. K. Dal, M. Y. W. Ng, N. Alonso-Rodriguez, E. Scarpa, L. Rizzello, G. Battaglia, G. Griffiths, F. Fenaroli, *EBioMedicine* **2020**, *58*, 102902.
